# Dietary Gradient, *Blastocystis* Colonisation, and Gut Microbiota Ecology in Mexican University Students: An Exploratory Cross-Sectional Study

**DOI:** 10.3390/foods15193383

**Published:** 2026-09-23

**Authors:** Alejandra Méndez-Hernández, Claudia Muñoz-Yáñez, Faviel Francisco González-Galarza, Adria Imelda Prieto-Hinojosa, Janeth Oliva Guangorena-Gómez

**Affiliations:** 1 Instituto de Ciencia y Medicina Genómica, Torreón 27000, Mexico; alemendez.icm@gmail.com; 2 Faculty of Health Sciences, Juárez University of the State of Durango, Gómez Palacio 35050, Mexico; claudiamunoz_y@hotmail.com; 3 Department of Molecular Immunobiology, Center for Biomedical Research, Universidad Autonoma de Coahuila, Torreón 27000, Mexico; faviel.gonzalez@uadec.edu.mx (F.F.G.-G.); dra.adriaprieto@tec.mx (A.I.P.-H.); 4 School of Engineering and Sciences, Tecnologico de Monterrey, Torreón 27000, Mexico

**Keywords:** gut microbiota, dietary gradient, *Blastocystis*, alpha diversity, beta diversity, microbial ecology

## Abstract

Gut microbiota is shaped by interactions among diet, host characteristics, and microbial colonisation. This exploratory cross-sectional study examined associations between a dietary gradient, *Blastocystis* colonisation, and gut microbiota in 41 university students, seven (17.1%) of whom were colonised with *Blastocystis*. Dietary intake was assessed using a validated food frequency questionnaire, and a diet-ratio was derived from the relative energy contribution of industrialised and non-industrialised foods. Gut microbiota was characterised by 16S rRNA gene sequencing. The multivariable model including diet-ratio, age, and body mass index was associated with microbiota structure in the primary genus-level analysis (PERMANOVA: R^2^ = 0.090, *p* = 0.041), although this association was attenuated after adjustment for abdominal pain and was not retained using the de novo DADA2 ASV table. *Blastocystis* colonisation was not associated with overall microbiota composition but was associated with greater alpha diversity (Shannon: *p* = 0.033; Gini–Simpson: *p* = 0.043). These alpha-diversity associations remained statistically significant after additional adjustment for abdominal pain and genus-level rarefaction, although they were less consistent in other sensitivity analyses. Genus-level associations with the dietary gradient did not remain significant after false discovery rate correction. No statistically significant diet-ratio × *Blastocystis* interaction was detected, although the small number of colonised participants limited assessment of effect modification. Overall, these findings suggest that dietary and *Blastocystis*-related associations may involve different ecological dimensions of the gut microbiota and warrant confirmation in larger studies.

## 1. Introduction

The human gut microbiome constitutes a dynamic and highly diverse ecosystem that plays a fundamental role in host metabolism, immune function, and the maintenance of health [1,2,3,4]. The composition and diversity of this microbial community are dictated by an intricate interplay of environmental exposures, genetic factors, and lifestyle behaviours [5,6,7]. Although host genetics contribute to interindividual variation in the microbiota, evidence suggests that environmental and lifestyle factors account for a substantially larger proportion of observed variability [8].

Among the environmental determinants of the gut microbiota, diet is widely regarded as one of the most influential. Dietary habits can affect both the taxonomic composition and the ecological diversity of microbial communities [9,10]. In particular, dietary patterns characterised by different levels of food processing have been consistently associated with differences in gut microbiota composition [11,12,13]. Diets rich in fibre and minimally processed foods have generally been linked to greater microbial diversity and the enrichment of taxa associated with beneficial metabolic functions, whereas diets dominated by highly processed foods have been associated with reduced diversity and less favourable microbial profiles [14]. Nevertheless, although diet is recognised as a dominant ecological factor, its effects on the microbiota are usually moderate and are influenced by other host and environmental factors [4].

Beyond bacteria, intestinal eukaryotes have increasingly been recognised as important components of the gut ecosystem [15,16]. Among them, *Blastocystis* is one of the most frequently detected organisms in human populations worldwide [17]. Although historically considered a potential pathogen, the clinical and ecological significance of *Blastocystis* remains debated, with studies reporting associations ranging from gastrointestinal symptoms to apparently asymptomatic colonisation and greater microbial diversity [18]. Positive associations with bacterial alpha diversity, particularly the Shannon index, have been reported in several studies; however, these findings are not universal and may vary according to population and study context [19,20].

Previous studies have evaluated diet, *Blastocystis* carriage, and the gut microbiota within the same populations and have reported associations among these factors [14,21,22]. However, these studies have mainly examined diet and *Blastocystis* in parallel in relation to the microbiota. Direct evaluation of whether *Blastocystis* colonisation modifies the association between an individual-level dietary exposure and global gut microbiota structure has received limited attention. Examining diet and *Blastocystis* within the same analytical framework may therefore help clarify how each factor relates to different ecological dimensions of the bacterial community.

From an ecological perspective, diet and *Blastocystis* may influence distinct dimensions of gut microbiota structure. Diet is thought to primarily affect community composition (beta diversity) by modulating the relative abundance of microbial taxa [23], whereas *Blastocystis* colonisation has been associated with differences in bacterial alpha diversity, although the direction and consistency of these associations vary across populations and analytical approaches [19,24,25,26]. However, this ecological framework has received limited direct evaluation in human cohorts integrating individual-level dietary data, *Blastocystis* colonisation and microbiota profiling. Young adults represent a relevant population for investigating these ecological relationships because age is an important determinant of gut microbiota composition, and studying a relatively homogeneous cohort reduces variability associated with ageing and chronic disease [27,28].

Therefore, this study aimed to evaluate the associations of dietary gradient and *Blastocystis* colonisation with complementary dimensions of gut microbiota diversity and composition in a cohort of university students. We further examined whether *Blastocystis* colonisation modified the association between the dietary gradient and gut microbiota structure.

## 2. Materials and Methods

### 2.1. Study Population

This cross-sectional study was conducted in a cohort of university students. Participants were eligible if they were undergraduate students enrolled in the Medicine and Nutrition programmes at the Faculty of Health Sciences and provided written informed consent before participation. Students who reported antibiotic use during the three months preceding sampling were excluded. Participants were removed from the study if a faecal sample was insufficient or unavailable or if the questionnaires required by the original study protocol were incomplete. A total of 46 participants fulfilled the eligibility criteria, were enrolled in the study, and underwent stool sample collection for 16S rRNA gene sequencing. All 46 faecal samples were successfully sequenced and processed through the bioinformatic pipeline. During data curation, five participants were excluded because of incomplete dietary or metadata records, resulting in a final analytical sample of 41 participants. This resulted in a complete-case dataset for the subsequent multivariable analyses. Participants were recruited voluntarily and provided written informed consent prior to enrolment. Demographic, anthropometric, and dietary information was collected from all participants. The FACSA participants included in the present study were recruited as part of the same 2021 cohort previously reported in a combined FACSA–PAVILA study investigating diet quality, *Blastocystis* colonisation, and gut microbiota [29]. However, the present study differs substantially in both scope and analytical approach. Whereas the previous publication evaluated only *Blastocystis*-colonised participants using V3 16S rRNA gene sequencing and the Healthy Eating Index-2020 (HEI-2020), the present study included all eligible FACSA participants with complete dietary and microbiota data, irrespective of *Blastocystis* colonisation status, and generated a new microbiota dataset based on V3–V4 16S rRNA gene sequencing. The primary objective was to evaluate an ecological dietary gradient (diet-ratio) in relation to gut microbiota composition using compositional multivariable analyses. Other publications from the FACSA research programme addressed distinct research questions and are cited only where directly relevant [30,31].

### 2.2. Anthropometric Measurements and Dietary Assessment

#### 2.2.1. Anthropometric Assessment

Anthropometric measurements were obtained in person using standardised procedures. Body weight and body composition were assessed with a calibrated bioimpedance analyser (Omron HBF-514CLA; OMROM Healthcare Co, Ltd., Kyoto, Japan), whilst height was measured with a standardised stadiometer (Seca 213). Body mass index (BMI) was calculated as weight (kg) divided by height squared (m^2^) and was included as a covariate in the statistical analyses to account for potential effects on gut microbiota composition.

#### 2.2.2. Assessment of Dietary Intake

The dietary information of the university students was collected using a Food Frequency Questionnaire (FFQ) previously validated by Macedo et al. (2013) [32]. To improve the quality and consistency of dietary data collection, the questionnaire was administered face to face by trained dietitians, who recorded participants’ responses using digital forms (Google Forms).

Responses were exported to Microsoft Excel and processed using a custom script developed in Stata (StataCorp LLC, College Station, TX, USA), in which the reported consumption frequencies were converted into daily equivalents and subsequently into grams per day for each food item included in the FFQ.

The estimated grams per day were then imported into a predefined Evalfinut 2.0 template (Ibero-American Nutrition Foundation, FINUT) [33], where each food item was linked to its corresponding food composition code based on the Spanish Food Composition Database (BEDCA 2.0) [34] and the USDA National Nutrient Database for Standard Reference (Release 28) [35]. Evalfinut was used to estimate total energy intake and macro- and micronutrient intake for each participant.

The nutritional output generated by Evalfinut was subsequently exported back to Stata, where a custom script calculated the percentage contribution of each predefined food group to total energy intake (%E). Food groups were defined according to the structure of the validated FFQ (e.g., fruits, vegetables, whole grains, refined cereals, dairy products, animal-source foods, and industrialised foods). These energy contributions constituted the basis for constructing the dietary gradient (diet-ratio), allowing dietary intake to be characterised using an energy-adjusted dietary gradient based on the relative contribution of food groups rather than the intake of individual nutrients.

The plausibility of reported energy intake was assessed using the energy intake-to-basal metabolic rate ratio (EI:BMR), following the Goldberg and Black approach [36,37]. Basal metabolic rate (BMR) was estimated using the Schofield predictive equations [38], and physical activity level was assessed using the short version of the International Physical Activity Questionnaire (IPAQ-SF) [39]. Participants were classified as under-reporters, plausible reporters, or over-reporters according to established cut-off values. Consistent with current methodological recommendations, this classification was used to assess the plausibility of dietary reporting and was not applied as an exclusion criterion to avoid introducing additional selection bias [31,40].

#### 2.2.3. Construction of the Dietary Gradient (Diet-Ratio)

To evaluate the diet from an ecological perspective in relation to gut microbiota structure, the food groups, expressed as percentage energy contribution (%E), were reclassified into two contrasting dietary components according to the dietary framework developed for the present study.

To increase the nutritional resolution of the model, the cereal group was disaggregated according to its degree of refinement. Refined grains (RG) were assigned to the industrialised pattern, alongside pastries, beverages, fats, miscellaneous items, and alcohol. In contrast, whole grains (WG) were included in the non-industrialised pattern, together with fruits, vegetables, legumes, fish, eggs, dairy products, animal-source foods (ASF), and nuts.

Based on this classification, two continuous variables representing the summed energy contribution of the food groups assigned to the industrialised and non-industrialised components were calculated. These components were treated as distinct dietary dimensions rather than as complementary proportions of total energy intake. Pearson correlation analysis was performed to evaluate the relationship between the two components and to determine whether the diet-ratio primarily reflected either component alone. Because the two components differed in scale and distribution, both were standardised using z-score transformation (mean = 0, standard deviation = 1) to ensure comparability.

Subsequently, a continuous variable termed diet-ratio was constructed as the difference between the standardised industrialised and non-industrialised components (diet-ratio = industrialised_z − non-industrialised_z). The diet-ratio represents a one-dimensional energy-adjusted dietary gradient capturing the relative predominance of industrialised versus non-industrialised foods. Unlike traditional mathematical ratios, this approach avoids instability associated with values close to zero and highly skewed distributions, providing a continuous, symmetrical, and statistically suitable measure for multivariable analyses.

In this context, positive diet-ratio values indicate a greater relative contribution of industrialised foods, whereas negative values indicate a greater contribution of non-industrialised foods.

Finally, to facilitate interpretation of the ordination analyses, the diet-ratio was categorised into tertiles representing low, intermediate, and high levels of dietary industrialisation. This categorisation was used for visualisation in the distance-based redundancy analyses (db-RDA) and other exploratory analyses. A detailed description of the variables, calculations, tertile cutoff points, and sensitivity analyses supporting the construction of the dietary gradient is provided in Table A1 and Table A8.

### 2.3. Sample Collection and DNA Extraction

Faecal samples were collected by the participants in sterile containers and stored at −80 °C until processing. Total bacterial genomic DNA was extracted from 200 mg of faeces using the E.Z.N.A.^®^ Stool DNA Kit (OMEGA Bio-Tek, Inc., Norcross, GA, USA) according to the manufacturer’s instructions, including the initial bead-beating step for mechanical disruption prior to DNA purification. DNA integrity was verified by electrophoresis on a 1.5% agarose gel, and DNA concentration was determined using a NanoDrop spectrophotometer (Thermo Fisher Scientific Inc., Waltham, MA, USA).

### 2.4. Molecular Detection of Blastocystis

The detection of *Blastocystis* DNA in the faecal extracts was carried out using a real-time polymerase chain reaction (qPCR) assay with a specific probe. The primers F1/GGT CCG GTG AAC ACT TTG GAT TT and R1/CCT ACG GAA ACC TTG TTA CGA CTT CA [41] were used alongside a FAM/TCG TGT AAA/ZEN/TCT TAC CAT TTA GAG GA/3IABkFQ probe (Integrated DNA Technologies, IDT). The reactions were performed in a RotorGene^®^ thermocycler (Qiagen, Hilden, Germany) using the QuantiNova Probe RT-PCR Kit (Qiagen, Hilden, Germany) as a probe-based amplification master mix; no reverse-transcription step was performed because genomic DNA was the target. Thermocycling conditions were 95 °C for 5 min, followed by 40 cycles of 95 °C for 5 s and 60 °C for 30 s. Negative controls were included during DNA extraction and qPCR amplification, together with a positive amplification control. All samples were analysed in duplicate. The assay was used qualitatively to determine the presence or absence of *Blastocystis*. An analytical limit of detection was not determined in the present study.

#### *Blastocystis* Subtyping

*Blastocystis*-positive samples were further characterised by endpoint PCR using sequence-tagged site (STS) primers specific for subtypes ST1, ST2, ST3, ST4, ST5, and ST7, following previously described protocols [42]. PCR amplification was performed using subtype-specific primer sets [30], and the resulting amplicons were resolved by agarose gel electrophoresis. Subtypes were assigned according to the expected amplicon size. Human β-globin amplification was used as an internal control to verify DNA quality and PCR performance.

### 2.5. Gut Microbiota Profiling

For this analysis, gut microbiota was characterised using sequencing of the V3 and V4 hypervariable regions of the 16S rRNA gene using primers containing Illumina overlap adapters. Amplicons were purified with AMPure XP beads and subsequently indexed using the Nextera XT v2 kit to add dual indices. DNA concentrations were quantified using a Qubit 3.0 fluorometer (Thermo Fisher Scientific Inc., Waltham, MA, USA). Finally, the libraries were normalised, pooled in equimolar amounts, and sequenced on the Illumina MiSeq™ platform using the MiSeq^®^ V2 reagent kit (500 cycles) to generate 250 bp paired-end reads.

### 2.6. Bioinformatics Processing

The sequencing workflow included all 46 successfully sequenced FACSA samples, whereas downstream integrated statistical analyses were restricted to the 41 participants with complete microbiota, dietary, and anthropometric data. Raw paired-end 16S rRNA gene sequencing reads were processed in QIIME 2 version 2024.10.1 using the DADA2 plugin version 2024.10.0. Forward and reverse reads were denoised and merged using the denoise-paired method with truncation lengths of 240 bp for both reads, no 5′-end trimming (trim-left-f = 0; trim-left-r = 0), maximum expected errors of 2.0 for each read, a truncation quality score of 2, a minimum overlap of 12 bp, independent sample inference, and consensus-based chimera removal.

The 46 sequenced FACSA samples yielded 6,864,121 input reads. Per-sample input depth had a median of 152,396 reads (range: 88,884–184,330). Following quality filtering, 5,048,440 reads were retained (73.5% of input), with a per-sample median of 111,806 reads (range: 17,203–139,874). A total of 3,824,738 reads were successfully merged (55.7% of input); the median per-sample merge rate was 56.5% (range: 11.4–69.2%). After DADA2 consensus-based chimera removal, 438,699 reads remained, with a median of 9920 non-chimeric reads per sample (range: 1890–11,437). DADA2 inferred 28,390 ASVs across the 46 FACSA samples (Table A10).

The DADA2-derived ASVs were subsequently subjected to closed-reference clustering against the SILVA 138.2 SSU NR99 reference sequence database using VSEARCH v2.22.1 at 99% sequence identity. Sequences not meeting this threshold were classified as unmatched and excluded. An additional reference-based chimera-screening step was then performed using VSEARCH uchime-ref. Both borderline-retaining and no-borderline tables were generated during the original processing; the analyses reported in this study used the more stringent non-chimeric, no-borderline table. This final FACSA table contained 1482 reference-matched features before taxonomic aggregation. This closed-reference step was used in the original bioinformatic workflow to obtain a standardised SILVA-referenced feature table for downstream genus-level analyses; the de novo DADA2 ASV table was subsequently retained as a sensitivity analysis to assess the dependence of the findings on this processing choice.

Taxonomy was assigned using a Naive Bayes classifier trained against SILVA 138.2. Multiple sequence alignment was performed with MAFFT, and a phylogenetic tree was inferred using FastTree version 2.1.11. For the downstream statistical analyses reported here, the feature table was collapsed to the genus level, and taxa without genus-level assignment were excluded.

### 2.7. Bioinformatics and Statistical Analysis

Downstream statistical analyses were performed in R version 4.4.1 (R Foundation for Statistical Computing, Vienna, Austria), primarily using vegan version 2.7-1 together with additional packages for data manipulation, visualisation, and compositional analysis. The phyloseq package was not used in the analytical workflow presented in this study.

Alpha Diversity Assessment. Alpha diversity was calculated at the genus level from the complete, unfiltered, and unrarefied count matrix derived from the final reference-matched feature table; therefore, no rarefaction depth was applied to the primary analysis. Shannon diversity and the Gini–Simpson index (1 − D) were calculated using the diversity function in vegan. Differences in alpha diversity between participants colonised and non-colonised by *Blastocystis*, as well as among tertiles of the dietary gradient (diet-ratio), were evaluated using non-parametric tests (Wilcoxon rank-sum test for two-group comparisons and Kruskal–Wallis test for comparisons involving three groups). To address the primary objectives of the study, multivariable linear regression models were additionally fitted to evaluate the associations of *Blastocystis* colonisation, diet-ratio, age, body mass index (BMI), and sex with alpha diversity. As a sensitivity analysis, these multivariable linear regression models were refitted with abdominal pain as an additional covariate to evaluate whether the associations between *Blastocystis* colonisation and alpha diversity were retained after this additional adjustment. Shannon and Gini–Simpson diversity were considered the primary alpha-diversity outcomes, whereas observed richness was evaluated as a complementary sensitivity outcome. The prevalence filter excluding genera detected in 10% or fewer of samples was applied only before the beta-diversity and taxon-level compositional analyses and was not used for the calculation of alpha diversity.

Beta Diversity Analysis and Multivariate Modelling. Prior to beta-diversity analyses, bacterial genera detected in 10% or fewer of samples were excluded to reduce sparsity. A pseudocount of 1 was added to every entry in the prevalence-filtered count matrix before centred log-ratio (CLR) transformation; Bayesian-multiplicative replacement was not used. Euclidean distances calculated from the CLR-transformed matrix (Aitchison distances) were subsequently used to evaluate differences in gut microbial community composition. Permutational multivariate analysis of variance (PERMANOVA) was applied to assess the contribution of the dietary gradient (diet-ratio), *Blastocystis* colonisation, and host characteristics to microbial community variation. To address the primary study objective, the principal multivariable model included diet-ratio, age, and body mass index (BMI), representing the main dietary exposure and host-related covariates of interest. Additional models including sex, *Blastocystis* colonisation, and the diet-ratio × *Blastocystis* interaction were fitted to evaluate their potential contribution to gut microbiota structure. Statistical significance was assessed using 9999 permutations, with a fixed random seed (set.seed(123)) specified immediately before each permutation test.

To verify the assumptions of the multivariable analyses, homogeneity of multivariate dispersion among groups was evaluated using PERMDISP prior to PERMANOVA. In addition, variance inflation factors (VIFs) were calculated for all explanatory variables included in the multivariable models to assess potential multicollinearity. No evidence of heterogeneous dispersion or problematic multicollinearity was detected (Table A3).

#### 2.7.1. Distance-Based Redundancy Analysis (db-RDA)

Distance-based redundancy analysis (db-RDA) was performed using the same CLR-transformed Euclidean distance matrix to quantify the proportion of constrained variation explained by the explanatory variables and to visualise multivariable associations between dietary gradient, *Blastocystis* colonisation, and gut microbial composition. Statistical significance of the constrained ordinations was evaluated using Monte Carlo permutation tests. Species scores were extracted to identify bacterial genera contributing most strongly to the observed ecological gradients.

#### 2.7.2. Taxon-Level Correlation Analysis

Spearman rank correlations between the dietary gradient (diet-ratio) and bacterial genera were calculated using CLR-transformed abundances after prevalence filtering (>10% of samples). *p*-values were adjusted for multiple comparisons using the Benjamini–Hochberg false discovery rate (FDR) procedure.

#### 2.7.3. Sensitivity Analyses

Additional multivariable models were fitted including abdominal pain as an additional covariate to evaluate the stability of the primary multivariable associations. Smoking status was evaluated as a potential confounding variable but was not retained in the final multivariable models because of its low prevalence in the study population and the absence of an association with either *Blastocystis* colonisation or the diet-ratio. The potential confounding variables considered in this study and the rationale for their inclusion or exclusion are summarised in Table A4.

An additional sensitivity analysis was performed excluding participants classified as implausible energy reporters according to the Goldberg–Black method. Total energy intake, the dietary gradient (diet-ratio), and the primary multivariable models were re-evaluated after their exclusion to assess whether the inclusion of implausible reporters influenced the main findings. The results of these analyses are presented in Table A8. Statistical significance was defined as *p* < 0.05 for all analyses. Additional sequencing-depth and bioinformatic sensitivity analyses were conducted to assess whether the main diversity findings were sensitive to the closed-reference clustering step or to uneven sequencing depth. Key alpha- and beta-diversity analyses were repeated using the de novo DADA2 ASV table prior to closed-reference clustering, after exclusion of the lowest-depth sample (ESCTRL15; 1890 reads), and after rarefaction without replacement to 4250 reads per sample. For the rarefaction sensitivity analysis, the rarefied feature table was subsequently evaluated at the same genus-level resolution as the primary analysis to isolate the influence of sequencing depth while maintaining a comparable taxonomic representation. These analyses were conducted as sensitivity analyses and did not replace the prespecified genus-level analyses used as the primary analytical approach.

## 3. Results

### 3.1. Characteristics of the Study Population

The final analytical sample comprised 41 university students after data curation and quality control procedures. The mean age of the participants was 21.20 ± 1.35 years. The mean body mass index (BMI) was 25.10 ± 4.72 kg/m^2^, indicating that the cohort average was slightly above the conventional upper limit of the normal-weight range despite the young mean age of the participants, and was higher in men than in women. The mean body fat percentage was 34.96 ± 8.73%. The diet-ratio had a mean value of 0.000 ± 1.419 and ranged from −2.879 to 3.688. Regarding sex distribution, 26.8% of the participants were male, and 73.2% were female. Concerning *Blastocystis* colonisation, most participants were not colonised (82.9%, *n* = 34), whereas 17.1% (*n* = 7) were colonised. The plausibility of dietary reporting was also assessed. Most participants were classified as plausible reporters (35/41, 85.4%), whereas one participant (2.4%) was identified as an under-reporter and five participants (12.2%) as over-reporters. Baseline demographic, anthropometric, and participant characteristics are summarised in Table 1.

### 3.2. Dietary Gradient Based on Food Groups

The participants’ diet was characterised by the relative contribution of food groups to the total energy intake (%E). From this information, a continuous dietary gradient (diet-ratio) was constructed to reflect the relative predominance of industrialised foods versus non-industrialised foods. The diet-ratio showed a continuous distribution in the cohort (Figure 1), evidencing a dietary spectrum ranging from patterns dominated by non-industrialised foods (negative values) to those with a higher contribution of industrialised foods (positive values).

The industrialised and non-industrialised components were not correlated (Pearson’s *r* = −0.007, *p* = 0.965), indicating that they represented distinct dietary dimensions in this cohort. The diet-ratio was positively correlated with the industrialised component (*r* = 0.710, *p* < 0.001) and negatively correlated with the non-industrialised component (*r* = −0.710, *p* < 0.001).

To facilitate interpretation, the diet-ratio was categorised into tertiles defining low, intermediate, and high levels of dietary industrialisation. The lower and upper tertile cut-points were −0.520 and 0.159, respectively, yielding groups of 14, 13, and 14 participants (Table A8). This classification allowed the identification of a progressive transition in the dietary structure along the gradient, characterised by an increasing contribution of industrialised foods.

### 3.3. Global Structure of the Gut Microbiota

Principal coordinates analysis (PCoA), based on Euclidean distances calculated from CLR-transformed data, showed considerable variability in the composition of the gut microbiota among participants (Figure 2). Although individuals classified in the different tertiles of the dietary gradient showed some degree of overlap, the observed dispersion suggests heterogeneity in the microbial structure of the cohort. This variability justified the application of subsequent multivariate models to formally evaluate the association between the dietary gradient and gut microbiota composition.

### 3.4. Association Between the Dietary Gradient, Host Variables, and Gut Microbiota Structure

The PERMANOVA analysis showed that the reduced model including the dietary gradient (diet-ratio), age, and body mass index (BMI) was significantly associated with the global structure of the gut microbiota (R^2^ = 0.090, F = 1.22, *p* = 0.041). The same multivariable model was subsequently visualised using distance-based redundancy analysis (db-RDA), which showed a significant constrained ordination of the microbial community (F = 1.22, *p* = 0.041) (Figure 3).

In contrast, the complete model including age, sex, BMI, presence of *Blastocystis*, and *diet-ratio* did not show a statistically significant association (R^2^ = 0.139, F = 1.13, *p* = 0.097). Similarly, the model adjusted for *Blastocystis* was not statistically significant (PERMANOVA: R^2^ = 0.114, F = 1.16, *p* = 0.074; db-RDA: F = 1.16, *p* = 0.074).

The model including the interaction between the dietary gradient and *Blastocystis* colonisation was not statistically significant (PERMANOVA: R^2^ = 0.136, F = 1.10, *p* = 0.152; db-RDA: F = 1.10, *p* = 0.152).

To assess the robustness of the multivariable models, additional sensitivity analyses were performed by including abdominal pain as an additional covariate. After adjustment for abdominal pain, the associations observed in the reduced PERMANOVA and db-RDA models were no longer statistically significant. Abdominal pain itself was not significantly associated with gut microbial community composition or alpha diversity. In contrast, the positive associations between *Blastocystis* colonisation and alpha diversity remained statistically significant after additional adjustment for abdominal pain (Shannon, *p* = 0.033; Gini–Simpson, *p* = 0.040). These findings indicate that the beta-diversity association was sensitive to adjustment for abdominal pain, whereas the associations between *Blastocystis* colonisation and alpha diversity were retained under this additional covariate adjustment. Detailed results of the sensitivity analyses are presented in Table A2.

A second sensitivity analysis was performed excluding the six participants (14.6%) classified as implausible energy reporters according to the Goldberg–Black method. Although energy intake differed between plausible and implausible reporters (Wilcoxon *p* = 0.023), the distribution of the diet-ratio did not differ significantly (*p* = 0.252). Repeating the primary multivariable model after excluding implausible reporters produced similar estimates (PERMANOVA: R^2^ = 0.106, F = 1.22, *p* = 0.0382; db-RDA: adjusted R^2^ = 0.019, F = 1.20, *p* = 0.0382). The overall pattern was similar after exclusion of implausible energy reporters, indicating that the primary association was not driven by participants with implausible reported energy intake. (Table A8).

Marginal term-wise PERMANOVA analyses showed that no individual explanatory variable was significantly associated with gut microbiota structure in any of the multivariable models (Table A6). In the sensitivity model, abdominal pain was not significantly associated with microbial community composition (R^2^ = 0.018, F = 0.75, *p* = 0.921).

Homogeneity of multivariate dispersion was assessed using PERMDISP, and multicollinearity was evaluated using variance inflation factors (VIF). No significant differences in dispersion were detected among dietary groups, and all VIF values were below commonly accepted thresholds. Detailed PERMDISP and VIF results are presented in Table A3.

Additional sensitivity analyses were conducted to assess whether the primary beta-diversity finding was sensitive to sequencing depth and bioinformatic processing. Exclusion of the lowest-depth sample (ESCTRL15) did not materially alter the genus-level PERMANOVA result (R^2^ = 0.0926, F = 1.2243, *p* = 0.0358). When the de novo DADA2 ASV table prior to closed-reference clustering was analysed, the primary model was not statistically significant (R^2^ = 0.0827, F = 1.1117, *p* = 0.1264). In the rarefaction sensitivity analysis, in which the table was rarefied to 4250 reads per sample and evaluated at the same genus-level resolution as the primary analysis, the proportion of explained variation remained similar to that of the primary model (R^2^ = 0.0912, F = 1.2045), although the permutation test was marginally above the conventional significance threshold (*p* = 0.0537). These findings indicate that the magnitude of the genus-level association was largely preserved after controlling for sequencing depth, whereas statistical significance was less consistent when the microbiota was analysed using the de novo DADA2 ASV table (Table A11).

### 3.5. Relationship Between the Dietary Gradient and Gut Microbiota Structure

The db-RDA ordination (Figure 3) visually represents the multivariable association identified in the reduced model (diet-ratio, age, and BMI). Although considerable overlap was observed among dietary groups, the orientation of the explanatory vectors suggests that both the dietary gradient and host variables contribute to the distribution of samples in the constrained ordination space. These graphical findings are consistent with the results of the reduced multivariable model presented in Table 2.

To evaluate whether these associations were reflected in global diversity changes, alpha diversity indices were analysed across the different dietary gradient tertiles. No significant differences in alpha diversity were observed among the groups defined by the level of industrialised food consumption. Neither the Shannon index nor the Gini–Simpson index showed statistically significant differences (Kruskal–Wallis, *p* = 0.120 and *p* = 0.127, respectively) (Figure A2).

### 3.6. Role of Blastocystis as an Ecological Factor

The role of *Blastocystis* in relation to gut microbiota structure was evaluated using adjusted multivariable models. The model including *Blastocystis* alongside the dietary gradient (diet-ratio), age, and BMI was not statistically significant (PERMANOVA: R^2^ = 0.114, F = 1.16, *p* = 0.074; db-RDA: F = 1.16, *p* = 0.074). In the marginal term-wise analysis, *Blastocystis* colonisation was not significantly associated with gut microbiota structure.

To further characterise the *Blastocystis*-positive samples, subtype identification was performed using the STS-PCR approach. ST3 was detected either alone or in combination with ST5, whereas four samples could not be assigned to any of the targeted subtypes (Table A5).

### 3.7. Interaction Between the Dietary Gradient and Blastocystis

The interaction analysis between the dietary gradient (diet-ratio) and the presence of *Blastocystis* showed no significant association with gut microbiota structure (overall interaction model PERMANOVA: *p* = 0.152; diet-ratio × *Blastocystis* term: *p* = 0.714; db-RDA: *p* = 0.152). These analyses provided no statistically detectable evidence of effect modification by *Blastocystis*; however, the small number of colonised participants limited the ability to evaluate the interaction reliably.

### 3.8. Taxa Associated with the Dietary Gradient

To explore specific associations between the dietary gradient (diet-ratio) and the gut microbiota, Spearman correlation analyses were performed at the genus level (Figure 4). Several nominal associations (*p* < 0.05 uncorrected) were identified between the diet-ratio and distinct bacterial taxa. Negative correlations were observed with genera such as *Flavonifractor*, *[Ruminococcus]_gnavus_group*, *Bacteroides*, *Ruthenibacterium*, *Paraprevotella*, *Streptococcus* and *Lachnospiraceae_UCG-010*, suggesting a higher relative abundance of these taxa in individuals with a lower degree of dietary industrialisation. Conversely, genera such as *UCG-005* and *NK4A214_group* showed positive correlations, indicating a potential association with more industrialised diets. Nevertheless, none of these associations remained significant following false discovery rate (FDR) correction for multiple comparisons (Table A9). Therefore, the genus-level findings should be considered exploratory and do not provide evidence of robust associations between specific bacterial genera and the dietary gradient.

### 3.9. Alpha Diversity and Blastocystis Colonisation

The alpha diversity of the gut microbiota was evaluated according to *Blastocystis* colonisation using the Shannon and Gini–Simpson indices (Figure 5). Colonised participants showed higher alpha diversity than non-colonised participants. Median Shannon diversity was 2.844 (IQR: 2.748–2.885) in colonised participants and 2.510 (IQR: 2.232–2.780) in non-colonised participants (Wilcoxon, *p* = 0.033). Median Gini–Simpson diversity was 0.891 (IQR: 0.880–0.897) and 0.837 (IQR: 0.769–0.891), respectively (*p* = 0.043). In multivariable linear regression models adjusted for diet-ratio, age, body mass index, and sex, *Blastocystis* colonisation remained positively associated with both Shannon (β = 0.398, 95% CI: 0.074–0.722, *p* = 0.018) and Gini–Simpson diversity (β = 0.075, 95% CI: 0.011–0.139, *p* = 0.023). Detailed descriptive statistics, effect sizes, adjusted models, and leave-one-out sensitivity analyses are presented in Table A7.

Sensitivity analyses were also performed to assess whether the alpha-diversity findings were influenced by sequencing depth or bioinformatic processing. After exclusion of the lowest-depth sample (ESCTRL15), the association remained statistically significant for Shannon diversity (*p* = 0.0390), whereas the Gini–Simpson result was marginally above the conventional significance threshold (*p* = 0.0503). Using the de novo DADA2 ASV table, neither association reached statistical significance (Shannon, *p* = 0.0503; Gini–Simpson, *p* = 0.1822). In contrast, after rarefaction to 4250 reads per sample followed by analysis at the same genus-level resolution as the primary analysis, the associations with *Blastocystis* colonisation remained statistically significant for both Shannon (*p* = 0.0299) and Gini–Simpson diversity (*p* = 0.0462) (Table A11).

Additionally, the association between *Blastocystis* colonisation and the global structure of the gut microbiota was evaluated using beta diversity analyses. No significant differences were observed in microbial composition between colonised and non-colonised individuals (Figure A1; PERMANOVA, *p* > 0.05). These results suggest that the presence of *Blastocystis* is not associated with detectable changes in the global structure of the microbial community.

## 4. Discussion

The present study investigated the relationship between the dietary gradient, *Blastocystis* colonisation, and gut microbiota structure in a cohort of young adults. Overall, the findings identified associations of dietary, host-related, and *Blastocystis*-related factors with different measures of gut microbial ecology; however, several of these associations were sensitive to analytical specification and should be considered exploratory.

### 4.1. Global Structure of the Microbiota and the Role of Diet

The reduced multivariable model, which included the dietary gradient (diet-ratio), age, and body mass index (BMI), was significantly associated with the overall structure of the gut microbiota. However, diet-ratio alone did not explain the observed variation. This finding supports the concept that gut microbial communities are influenced by multiple host and environmental factors acting together rather than by a single exposure [4].

The primary genus-level model identified an association between the dietary gradient, age, BMI, and overall gut microbiota structure. However, no individual variable showed a statistically significant marginal contribution to community variation. Moreover, the overall association was attenuated after adjustment for abdominal pain and was not statistically significant when the de novo DADA2 ASV table was analysed, indicating sensitivity to model specification and feature-table representation. In contrast, after rarefaction to 4250 reads per sample at the same genus-level resolution as the primary analysis, the proportion of explained variation remained essentially unchanged, although the permutation test was marginally above the conventional significance threshold. Previous studies have suggested that industrialised dietary patterns may affect the gut environment through several mechanisms, including changes in nutrient availability and intestinal barrier function [12]. In the present cohort, however, the observed beta-diversity association was modest and should be interpreted as exploratory, particularly given its sensitivity to covariate adjustment and alternative feature-table processing.

The present study complements our previous work while addressing a distinct research question. Although it included participants from the same 2021 FACSA cohort described previously [29], it used a newly generated V3–V4 microbiota dataset and a different analytical framework. Unlike the previous study, which focused exclusively on *Blastocystis*-colonised individuals and diet quality assessed using the Healthy Eating Index-2020 (HEI-2020), the present work investigated ecological associations between a continuous dietary gradient, *Blastocystis* colonisation, and multiple dimensions of gut microbiota diversity across the complete FACSA cohort. Importantly, the inclusion of both colonised and non-colonised participants also allowed us to directly examine whether *Blastocystis* colonisation modified the association between the dietary gradient and overall gut microbiota structure, a question that could not be addressed in our previous study.

### 4.2. Taxa-Level Associations

Correlation analyses identified several nominal associations between the dietary gradient and bacterial genera, including negative correlations with *Bacteroides* and *Flavonifractor* and positive correlations with UCG-005 and NK4A214_group. However, none remained statistically significant after false discovery rate correction. Therefore, these taxon-level findings should be considered exploratory and do not support robust associations between the dietary gradient and specific bacterial genera in this cohort. These nominal associations may nevertheless be considered in the context of the VANISH (Volatile or Associated Negatively with Industrialised Societies of Humans) and BIoSSUM (Bloom or Selected in Societies of Urbanisation/Modernisation) frameworks. In Mexican populations, Bacteroidaceae and Verrucomicrobiaceae have been described among BIoSSUM taxa associated with urbanisation, whereas several taxa characteristic of traditional lifestyles have been classified as VANISH [43]. In the present cohort, however, *Bacteroides* and *Akkermansia* showed nominal negative correlations with the dietary gradient, whereas UCG-005 showed a positive correlation, indicating that taxon-level responses did not uniformly follow patterns expected from broader industrialisation-associated microbiome signatures. Such discrepancies may reflect differences in dietary exposures, population context, microbial ecological interactions, and taxon-specific responses to dietary substrates, consistent with evidence that gut microbial composition and individual taxa may respond differently to dietary exposures across experimental and population settings [9,44,45,46]. Importantly, none of these associations remained significant after FDR correction; therefore, these comparisons should be regarded as descriptive and hypothesis-generating rather than as evidence supporting or contradicting the VANISH/BIoSSUM framework.

### 4.3. Role of Blastocystis as an Ecological Modulator

The role of *Blastocystis* in the ecology of the gut microbiota remains controversial [25,26,47]. In the present study, *Blastocystis* colonisation was not associated with significant changes in the overall microbial community structure, as shown by the beta diversity analyses (Figure A1). However, colonised individuals presented higher alpha diversity according to both the Shannon and Gini–Simpson indices (Figure 5). This association remained after adjustment for diet-ratio, age, body mass index, and sex in multivariable linear regression models (Table A7). Nevertheless, these findings should be interpreted cautiously because only seven participants were colonised with *Blastocystis*, and the leave-one-out sensitivity analysis indicated that statistical significance was influenced by individual observations despite the direction of the association remaining consistent.

The prevalence of *Blastocystis* colonisation in the present cohort (17.1%) falls within the broad range reported across human populations and is comparable to the 19% prevalence reported by Stensvold et al. in a population-based study [48]. Differences in prevalence across studies may reflect population characteristics, environmental exposures, hygiene and sanitation conditions, and methodological differences in detection. Because recruitment for the present study occurred in 2021, during the COVID-19 pandemic, pandemic-related changes in social contact, mobility, and hygiene practices could also have influenced exposure patterns; however, their specific effect on *Blastocystis* prevalence cannot be determined from our cross-sectional data.

The higher alpha diversity observed among colonised participants is consistent with previous studies reporting greater bacterial diversity in individuals colonised by *Blastocystis* [24,25]. Several authors have proposed that *Blastocystis* may be associated with a more diverse intestinal ecosystem [17,20,24]. However, the present cross-sectional data cannot establish the direction of this relationship. *Blastocystis* colonisation may be associated with changes in the bacterial community, but the alternative explanation that individuals with intrinsically more diverse microbial communities provide a more permissive ecological environment for colonisation is equally plausible.

Importantly, the association between *Blastocystis* colonisation and higher alpha diversity was retained after additional adjustment for abdominal pain, with both Shannon and Gini–Simpson diversity remaining statistically significant. Abdominal pain itself was not significantly associated with either alpha-diversity index. This sensitivity analysis therefore suggests that the observed association between *Blastocystis* colonisation and alpha diversity was not explained by the presence of abdominal pain in this cohort. Nevertheless, this finding should be interpreted cautiously given the small number of colonised participants and the sensitivity observed in the leave-one-out and alternative feature-table analyses.

Potential metabolic interactions between *Blastocystis* and members of the gut microbiota have been proposed, including effects on tryptophan metabolism and microbial cross-feeding [20]. However, these mechanisms were not evaluated in the present study and therefore cannot be inferred from the observed association with alpha diversity.

Different *Blastocystis* subtypes may exhibit distinct biological and ecological characteristics [18]. In the present study, ST3 was detected either alone or in combination with ST5 among the samples that could be assigned to a subtype. However, four of the seven *Blastocystis*-positive samples could not be assigned to any of the subtypes targeted by the STS-PCR assay (ST1, ST2, ST3, ST4, ST5, and ST7). Given the small number of colonised participants and incomplete subtype assignment, the present data do not support subtype-specific ecological inference, and no subtype-specific analyses were performed.

### 4.4. Integrated Ecological Interpretation and Interaction Analysis

One of the main objectives of this study was to evaluate whether *Blastocystis* colonisation modified the association between the dietary gradient and gut microbiota structure. No statistically significant diet-ratio × *Blastocystis* interaction was detected. However, given the small number of colonised participants, this analysis had limited ability to detect effect modification, and the absence of a statistically significant interaction should not be interpreted as evidence that the dietary gradient and *Blastocystis* act independently.

In this cohort, dietary gradient and *Blastocystis* colonisation showed associations with different measures of gut microbial ecology. The primary model including the dietary gradient, age, and BMI was associated with community composition (beta diversity), although this association was sensitive to analytical specification. In contrast, *Blastocystis* colonisation was associated with greater within-sample diversity (alpha diversity), although this association should also be interpreted cautiously because of the small number of colonised participants and its sensitivity to individual observations.

Taken together, these exploratory findings suggest that dietary gradient and *Blastocystis* colonisation may relate to different measures of gut microbial ecology in this cohort of young adults. Larger studies with sufficient numbers of colonised participants will be required to determine whether *Blastocystis* modifies diet–microbiota associations.

### 4.5. Limitations

This study has several limitations that should be considered when interpreting the findings. First, the relatively small sample size (n = 41), including only seven participants colonised with *Blastocystis*, may have limited the statistical power to detect modest associations and reduced the precision of the estimated effects. Consequently, non-significant findings should not be interpreted as evidence of the absence of an effect but rather as reflecting the limited power of the study to detect small or moderate associations. Similarly, the non-significant PERMDISP results should be interpreted cautiously, as the modest sample size and unbalanced group sizes may have limited the power to detect differences in multivariate dispersion. Consistent with this limitation, the leave-one-out sensitivity analysis showed that the direction of the association between *Blastocystis* colonisation and alpha diversity remained positive, although statistical significance was sensitive to the removal of individual colonised participants. Additional sensitivity analyses also indicated some dependence of the beta-diversity findings on analytical specification. In particular, the primary model was not statistically significant when the de novo DADA2 ASV table was analysed, whereas rarefaction to 4250 reads per sample at the same genus-level resolution preserved a similar proportion of explained variation but yielded a permutation *p*-value marginally above the conventional significance threshold. These findings suggest that the observed beta-diversity association was more sensitive to feature-table representation than to sequencing-depth standardisation and should therefore be interpreted cautiously. Likewise, the absence of a statistically significant interaction between the diet-ratio and *Blastocystis* colonisation should not be interpreted as evidence that these factors act independently. Given the small number of colonised participants, the interaction analysis was substantially underpowered, and larger studies will be required to evaluate potential effect modification reliably. In addition, the cross-sectional design does not allow causal inferences regarding the relationships between dietary gradient, *Blastocystis* colonisation, and gut microbiota composition. Therefore, the observed associations should be interpreted cautiously and confirmed in larger, preferably longitudinal, studies.

Potential confounding factors were also considered. Sensitivity analyses including abdominal pain attenuated the association observed in the reduced beta-diversity model, which was no longer statistically significant after this additional adjustment. In contrast, the positive associations between *Blastocystis* colonisation and Shannon and Gini–Simpson diversity remained statistically significant after adjustment for abdominal pain, while abdominal pain itself was not significantly associated with either alpha-diversity index. Thus, the influence of abdominal pain differed across ecological outcomes: the beta-diversity finding was sensitive to this covariate adjustment, whereas the *Blastocystis*–alpha-diversity associations were retained. Nevertheless, these alpha-diversity findings should not be considered uniformly robust because their statistical significance remained sensitive to individual colonised participants and to alternative feature-table processing. Smoking was evaluated but was not retained in the final models because of its low prevalence in the study population. Alcohol intake was considered part of the dietary exposure because it contributed to the construction of the diet-ratio, and separate adjustment could have resulted in overadjustment. Physical activity was assessed using three Physical Activity Level (PAL) categories exclusively to estimate total energy expenditure within the Goldberg–Black method for identifying implausible energy reporters rather than as a quantitative measure of habitual physical activity. Consequently, it was not included as an independent covariate in the multivariable ecological models. Participants reporting antibiotic use within the previous three months were not eligible for enrolment according to the predefined study criteria. However, information on probiotic consumption and Bristol Stool Scale scores was not collected; therefore, residual confounding related to gut physiology and intestinal transit cannot be completely excluded. In addition, *Blastocystis* detection was based on a single stool sample, and intermittent shedding may have resulted in the misclassification of some colonised individuals. Another limitation was the incomplete subtype assignment among *Blastocystis*-positive samples. Four of the seven colonised participants could not be assigned to the ST1–ST5/ST7 panel. This could reflect the presence of *Blastocystis* subtypes not included in the ST1–ST5/ST7 STS-PCR panel, such as ST6 or ST8, reduced amplification efficiency associated with low template DNA concentrations, or other technical limitations inherent to subtype-specific PCR assays. Because SSU rRNA barcoding was not performed, these possibilities could not be distinguished. Furthermore, the original Rotor-Gene Q analysis was exported as an end-point assay, and quantitative amplification data (Ct values) were therefore not available for retrospective assessment of parasite load.

Despite these limitations, the study has several strengths. Dietary intake was assessed through face-to-face interviews using a food frequency questionnaire developed and validated in Mexican university students, a population comparable to that included in the present study. This provided an appropriate assessment of habitual dietary intake for our study population. In addition, gut microbiota composition was characterised by high-throughput sequencing of the V3–V4 regions of the 16S rRNA gene, and the analytical approach integrated dietary, anthropometric, demographic, and parasitological variables within multivariable ecological models. Together, these methodological features allowed dietary gradient and *Blastocystis* colonisation to be evaluated within the same analytical framework, including an explicit assessment of whether *Blastocystis* colonisation modified the association between the dietary gradient and overall gut microbiota structure.

## 5. Conclusions

This study examined the relationships between dietary gradient, age, body mass index, *Blastocystis* colonisation, and gut microbiota composition in a cohort of Mexican university students. The findings suggest that dietary gradient and *Blastocystis* colonisation may be associated with different ecological dimensions of the gut microbiota, although the observed associations showed varying sensitivity to analytical specification. In the primary genus-level analysis, the dietary gradient together with age and BMI was associated with gut microbial community structure, although this finding should be considered exploratory because it was attenuated after adjustment for abdominal pain and was not retained when the de novo DADA2 ASV table was analysed. Rarefaction at the same genus-level resolution preserved a similar proportion of explained variation, although statistical significance was marginal. *Blastocystis* colonisation was associated with greater alpha diversity, and the associations with Shannon and Gini–Simpson diversity remained statistically significant after additional adjustment for abdominal pain and after genus-level rarefaction but showed less consistency in the de novo DADA2 and leave-one-out sensitivity analyses. No statistically significant diet-ratio × *Blastocystis* interaction was detected; however, the small number of colonised participants precluded a reliable assessment of effect modification. Overall, these findings suggest that dietary and *Blastocystis*-related associations may be reflected in different ecological dimensions of the gut microbiota, but their magnitude and robustness require confirmation in larger and more diverse populations using longitudinal designs.

## Figures and Tables

**Figure 1:**
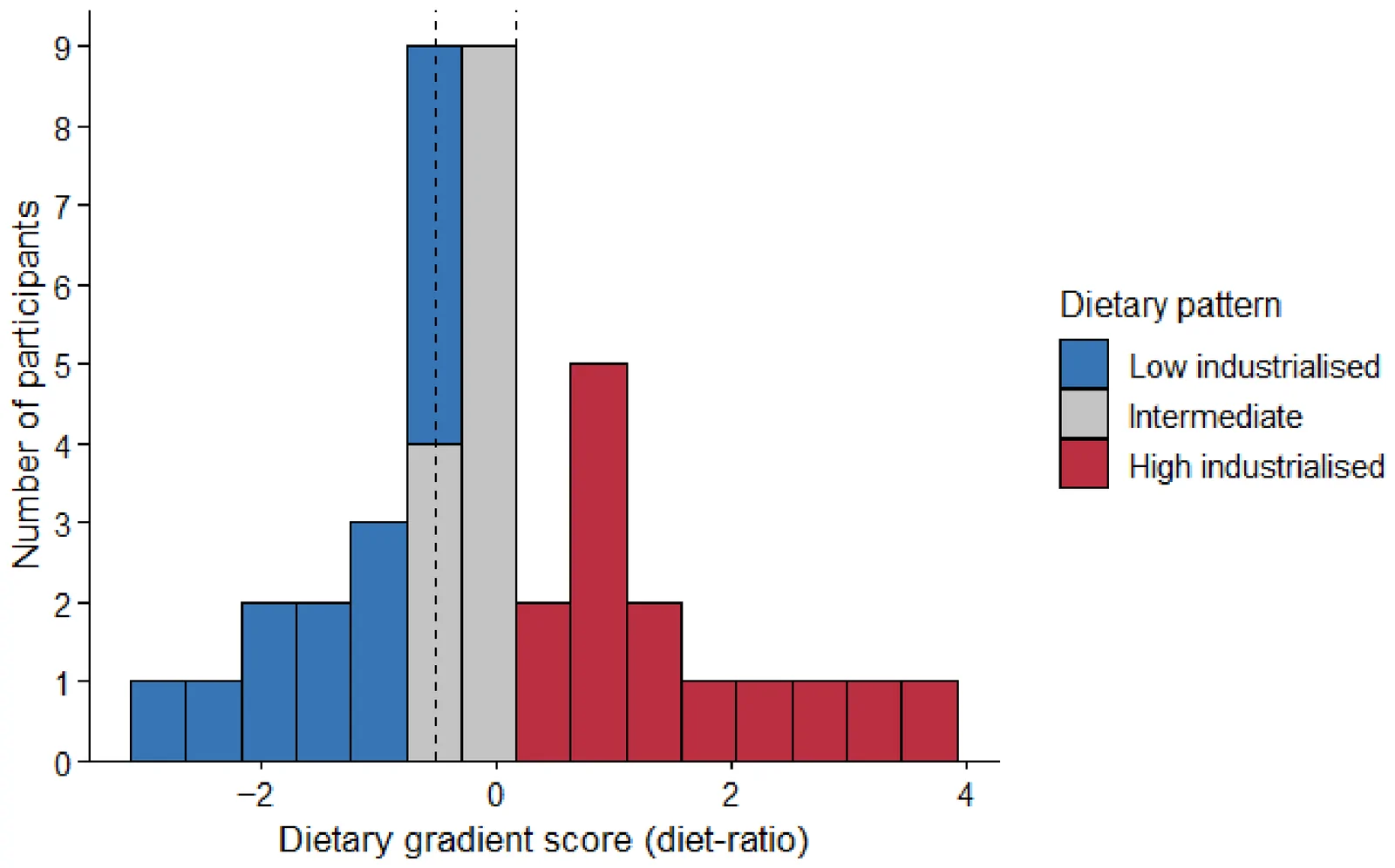
Distribution of the dietary gradient (diet-ratio) in the study population. Vertical dashed lines indicate the tertile cut-points (−0.520 and 0.159), defining the low industrialised (*n* = 14), intermediate (*n* = 13), and high industrialised (*n* = 14) dietary groups.

**Figure 2:**
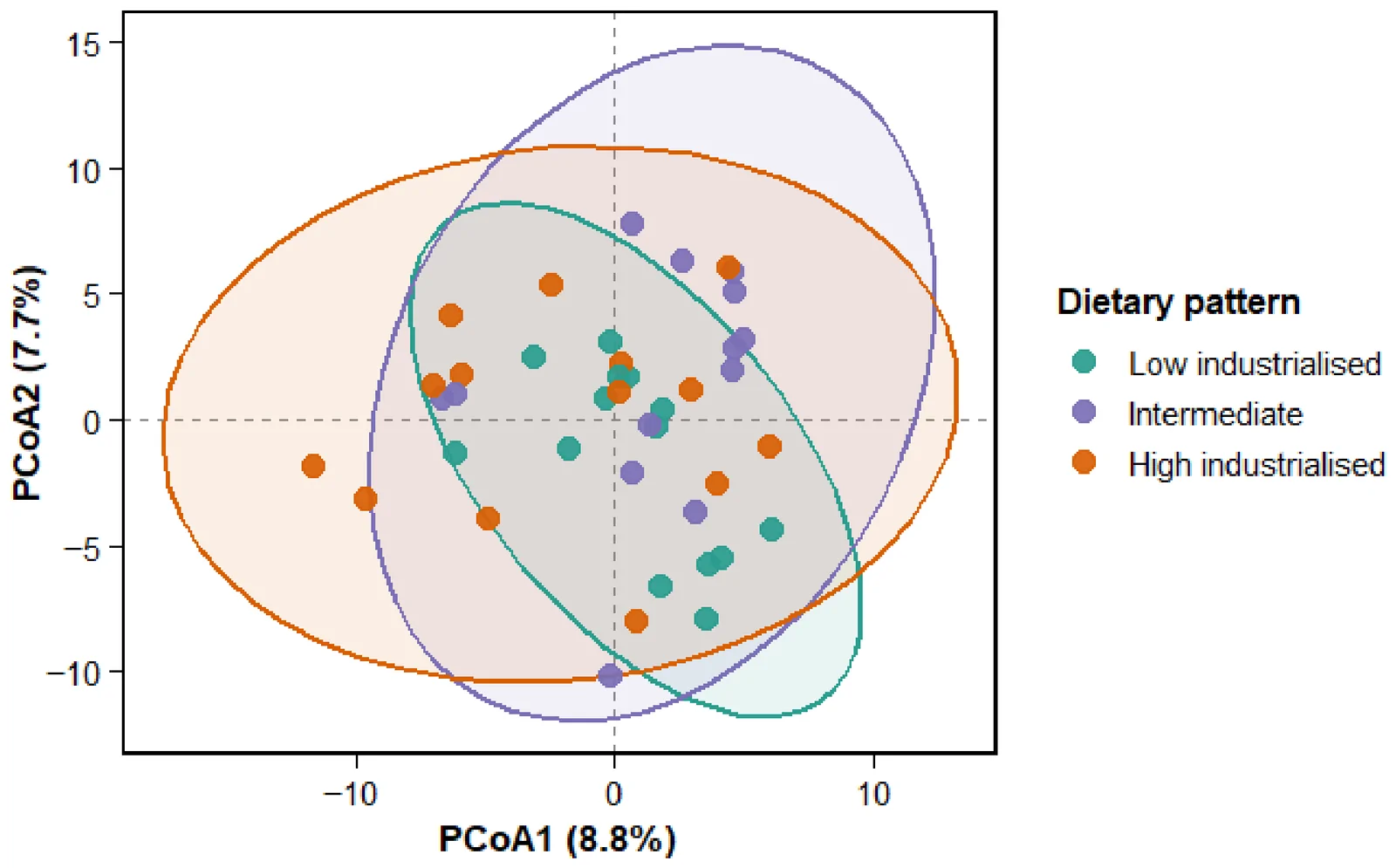
Global structure of the gut microbiota according to dietary gradient. The PCoA was performed based on Euclidean distances calculated from CLR-transformed microbiota data. Each point represents a participant, and colours indicate the tertiles of the dietary gradient. The ellipses show the dispersion of each dietary group. Ellipses represent 95% normal-theory data ellipses for each dietary group.

**Figure 3:**
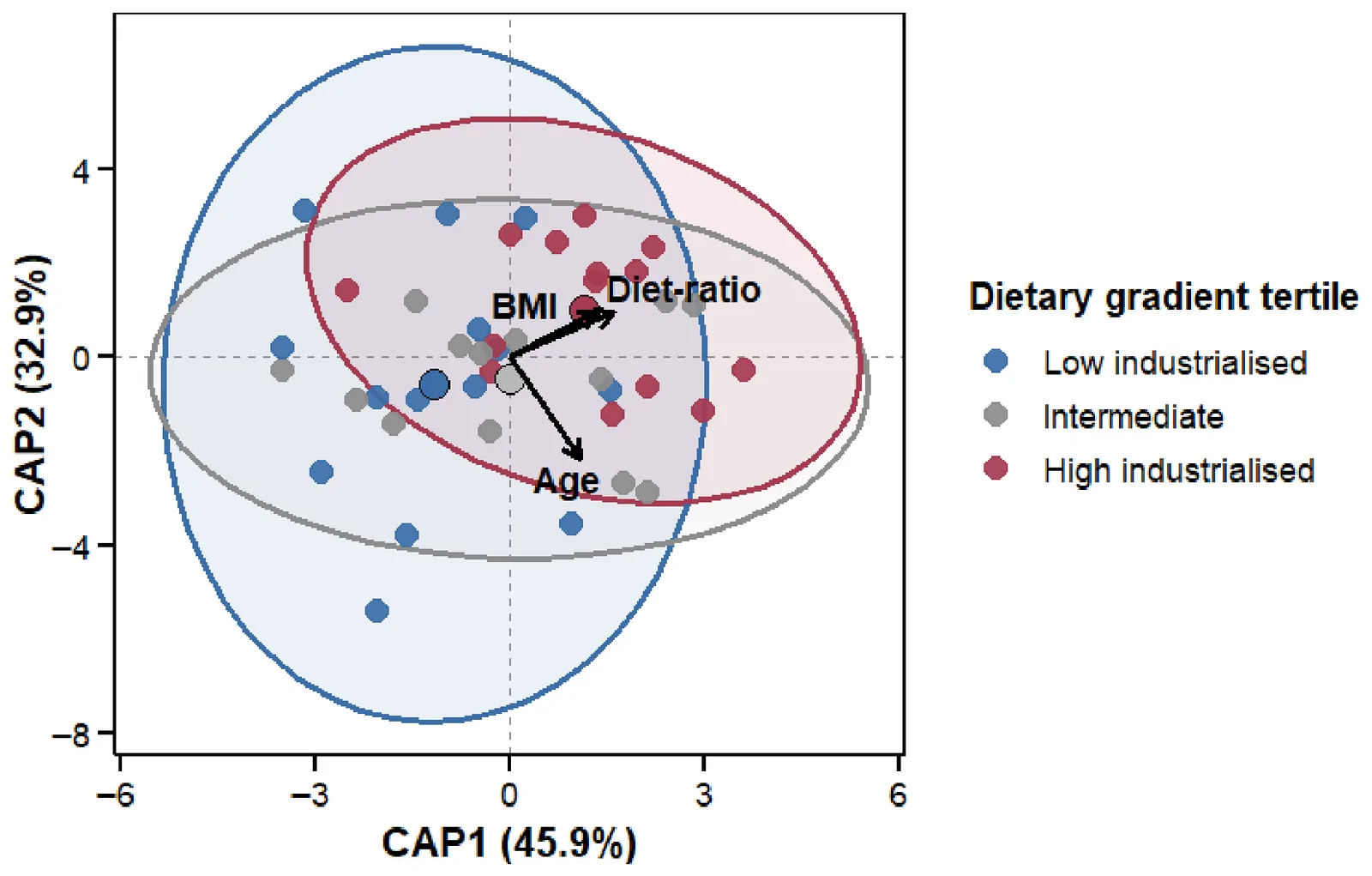
Distance-based redundancy analysis (db-RDA) of gut microbiota composition for the reduced multivariable model including diet-ratio, age, and BMI. The analysis was based on Euclidean distances calculated from CLR-transformed genus-level microbiota data. Each point represents one participant, with colours indicating tertiles of the dietary gradient used for visualisation (low, intermediate, and high industrialisation). Ellipses represent 95% normal-theory data ellipses for each dietary-gradient tertile. Black arrows represent the fitted explanatory-variable vectors for diet-ratio, age, and BMI in the constrained ordination space; their direction indicates the orientation of each variable relative to the ordination axes. CAP1 and CAP2 account for 45.9% and 32.9% of the constrained variance, respectively.

**Figure 4:**
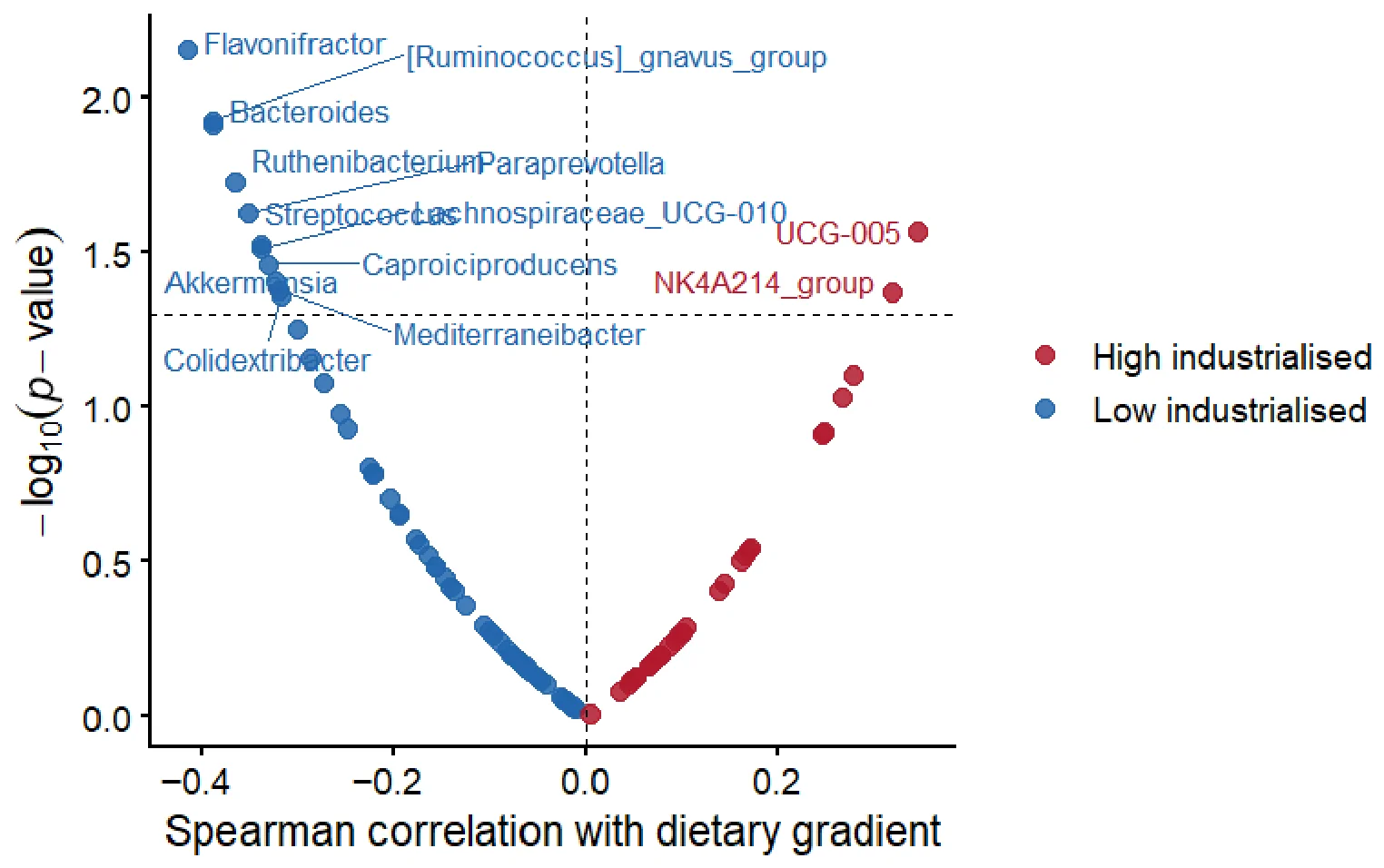
Associations between the dietary gradient (diet-ratio) and gut microbiota composition at the genus level. Each point represents a bacterial genus. The x-axis shows the Spearman correlation coefficient with the dietary gradient, while the y-axis represents the statistical significance expressed as −log10 (*p*-value). Genera positively associated with a higher industrialised dietary pattern are shown in red, whereas genera negatively associated (linked to lower industrialisation) are shown in blue. Dashed lines indicate the thresholds for correlation (ρ = 0) and nominal statistical significance (*p* = 0.05). Although several taxa exhibited nominal associations with the dietary gradient, none remained significant after false discovery rate correction.

**Figure 5:**
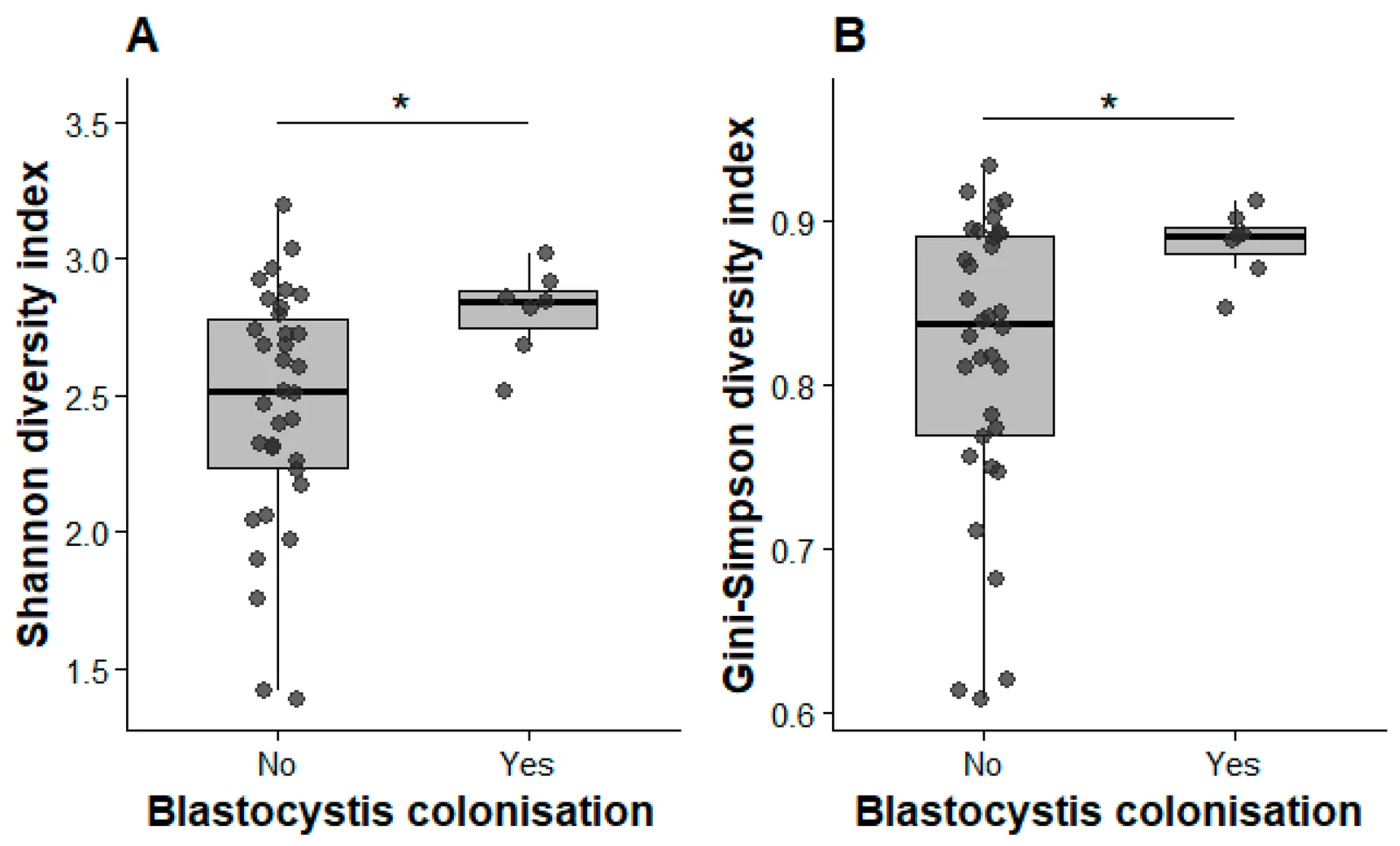
Alpha diversity of the gut microbiota according to *Blastocystis* colonisation status. Boxplots show Shannon diversity (**A**) and Gini–Simpson diversity (1 − D) (**B**) indices in colonised and non-colonised participants. Differences between groups were assessed using the Wilcoxon rank-sum test. Individual points represent participants, boxes show the interquartile range with the median indicated by the horizontal line, and the asterisk indicates a statistically significant difference between groups (*p* < 0.05).

**Table 1:** Characteristics of the study population.

Variable	Total *n* = 41	Male *n* = 11	Female *n* = 30
BMI, mean ± SD (kg/m^2^)	25.1 ± 4.72	27.19 ± 4.62	24.34 ± 4.59
Body fat, mean ± SD	34.96 ± 8.73	28.59 ± 6.24	37.29 ± 8.40
Age, mean ± SD (years)	21.2 ± 1.35	21.55 ± 1.86	21.07 ± 1.11
Diet-ratio, mean ± SD	0.000 ± 1.419	−0.184 ± 1.777	0.068 ± 1.293
*Blastocystis* colonisation, *n* (%)			
Non-colonised	34 (82.93%)	10 (90.91%)	24 (80%)
Colonised	7 (17.07%)	1 (9.09%)	6 (20%)
Smoking, *n* (%)			
No	35 (85.37%)	10 (90.91%)	25 (83.33%)
Yes	6 (14.63%)	1 (9.09%)	5 (16.67%)
Abdominal pain, *n* (%)			
No	17 (41.46%)	7 (63.64%)	10 (33.33%)
Yes	24 (58.54%)	4 (36.36%)	20 (66.67%)

BMI: body mass index; ±SD: standard deviation; (kg/m^2^): kilogram per square meter; *n*: frequency; (%): percentage. The observed diet-ratio ranged from −2.879 to 3.688. Values are presented as mean ± standard deviation or *n* (%), as appropriate.

**Table 2:** Multivariate models of gut microbiota structure.

Analysis	Model	df	R^2^	F	*p*-Value
PERMANOVA	Complete model: age + sex + BMI + *Blastocystis* + diet-ratio	5	0.139	1.13	0.097
PERMANOVA	Reduced model: diet-ratio + age + BMI	3	0.090	1.22	**0.041**
PERMANOVA	*Blastocystis*-adjusted model: *Blastocystis* + diet-ratio + age + BMI	4	0.114	1.16	0.074
PERMANOVA	Interaction model: diet-ratio × *Blastocystis* + age + BMI	5	0.136	1.10	0.152
db-RDA	Reduced model: diet-ratio + age + BMI	3	N/A	1.22	**0.041**
db-RDA	*Blastocystis*-adjusted model: *Blastocystis* + diet-ratio + age + BMI	4	N/A	1.16	0.074
db-RDA	Interaction model: diet-ratio × *Blastocystis* + age + BMI	5	N/A	1.10	0.152

Multivariate analyses evaluating the association between dietary gradient, host-related variables, *Blastocystis* colonisation, and gut microbiota structure. PERMANOVA and db-RDA analyses were performed using Euclidean distances calculated from CLR-transformed microbiota data. Statistically significant results (*p* < 0.05) are shown in bold. R^2^ values are reported for PERMANOVA, whereas db-RDA results are summarised using the permutation-test F statistic and *p*-value. N/A, not applicable.

**Figure A1:**
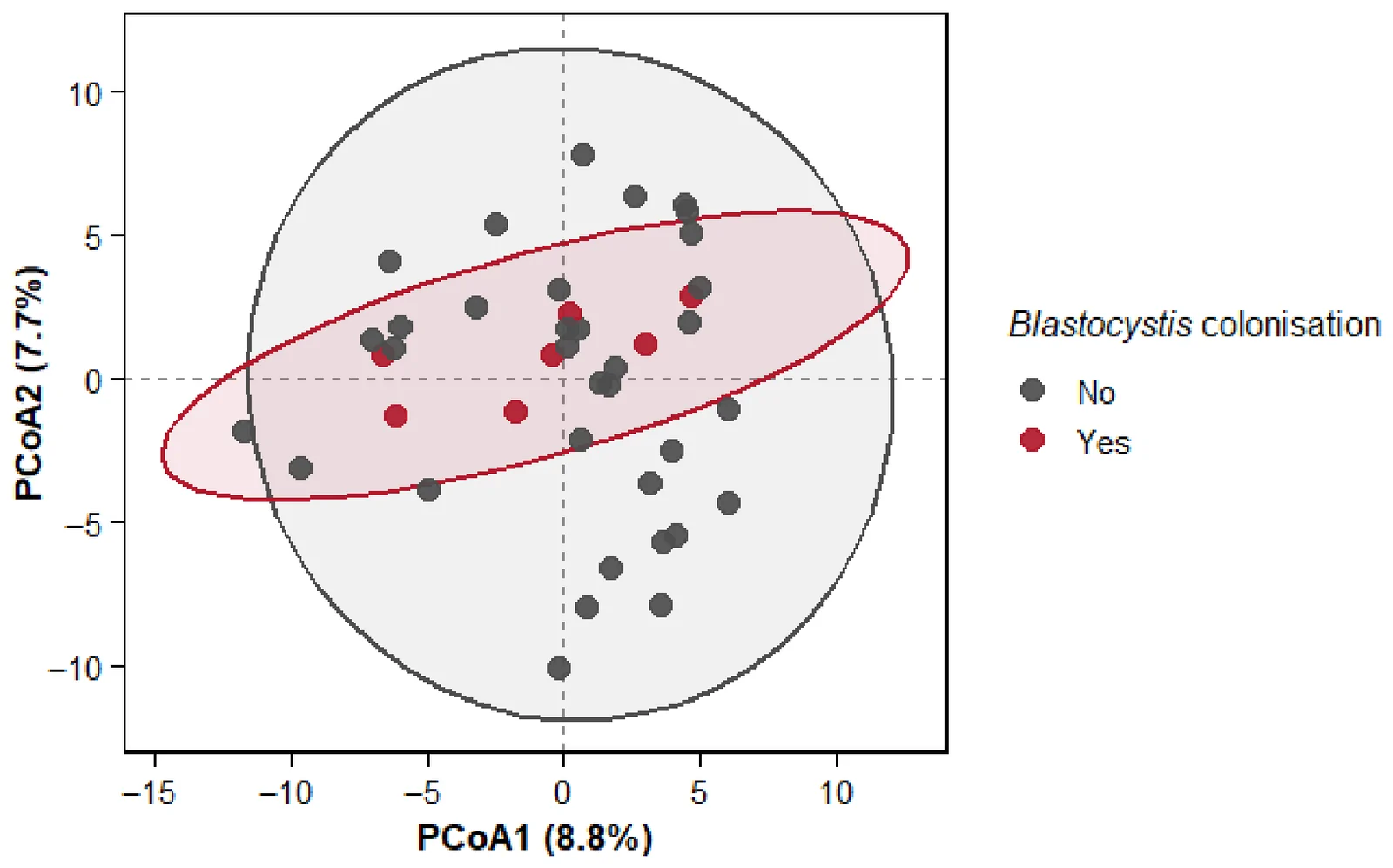
Principal coordinates analysis of gut microbiota composition according to *Blastocystis* colonisation status. PCoA was based on Euclidean distances calculated from centred log-ratio-transformed genus-level abundance data. Each point represents one participant. Ellipses represent 95% normal-theory data ellipses for colonised and non-colonised groups.

**Figure A2:**
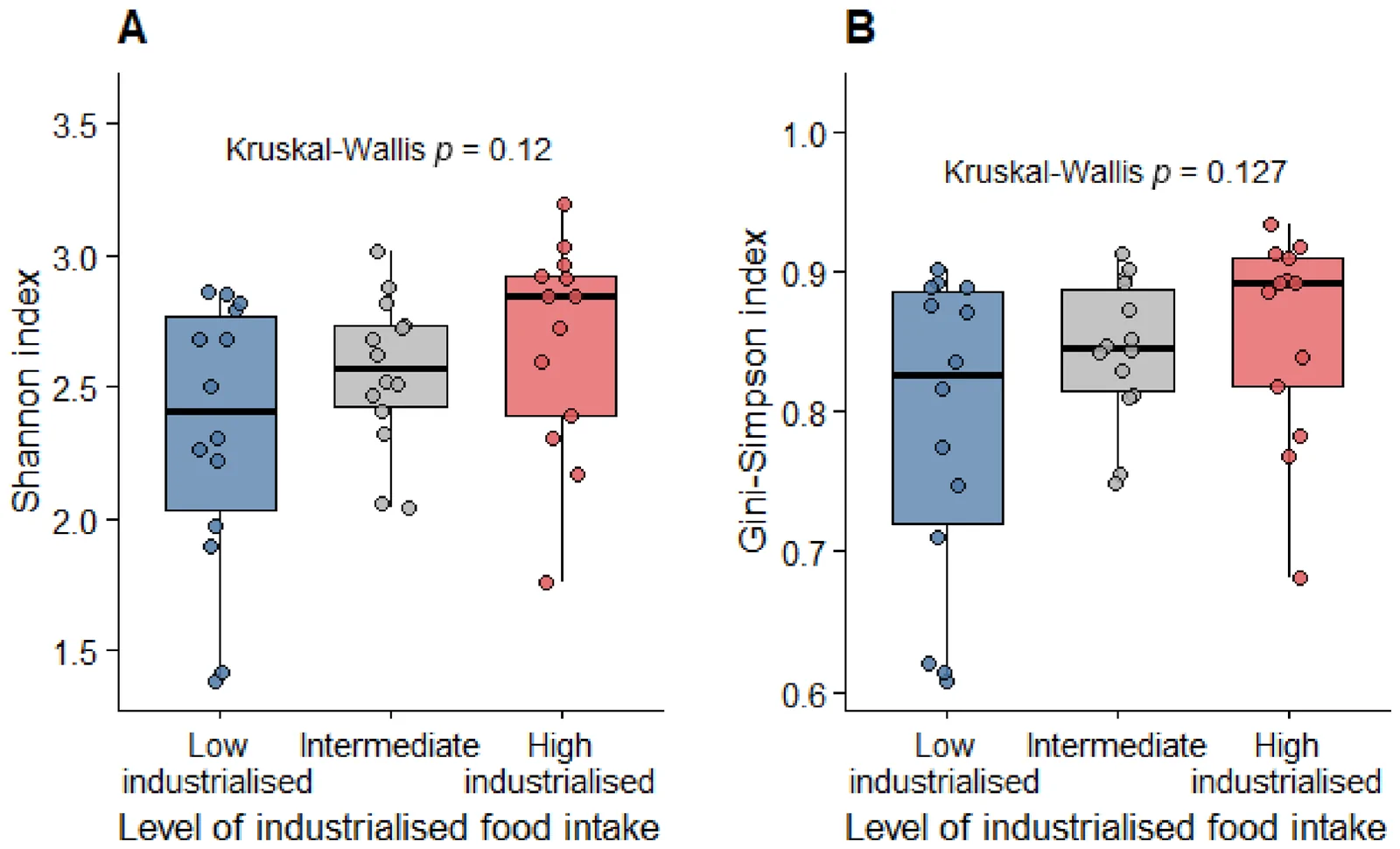
Alpha diversity of the gut microbiota according to dietary gradient defined by tertiles of industrialised food intake. Boxplots represent Shannon (**A**) and Gini–Simpson (1 − D) (**B**) indices. *p*-values were calculated using the Kruskal–Wallis test.Individual points represent participants. Boxplots show the distribution of diversity indices across the three levels of industrialised food intake (low, intermediate, and high), with the horizontal line within each box indicating the median. Colours correspond to the three dietary groups.

## Data Availability

The raw 16S rRNA gene sequencing data generated in this study have been deposited in the NCBI Sequence Read Archive (SRA) under BioProject accession number PRJNA1506799 (https://www.ncbi.nlm.nih.gov/bioproject/PRJNA1506799, accessed on 22 September 2026). Metadata supporting the findings of this study are available from the corresponding author upon reasonable request. Individual-level dietary and anthropometric data are not publicly available because of ethical and privacy restrictions.

## Abbreviations

The following abbreviations are used in this manuscript:

%E	Percentage of total energy
AhR	Aryl hydrocarbon receptor
ASF	Animal-sourced foods
ASVs	Amplicon sequence variants
BEDCA	Spanish Food Composition Database
BIoSSUM	Bloom or Selected in Societies of Urbanisation/Modernisation
BMI	Body mass index
CLR	Centred log-ratio
db-RDA	Distance-based redundancy analysis
FDR	False discovery rate
FFQ	Food frequency questionnaire
FINUT	Ibero-American Nutrition Foundation
IDT	Integrated DNA Technologies
PCoA	Principal coordinates analysis
PERMANOVA	Permutational multivariate analysis of variance
qPCR	Real-time polymerase chain reaction
RG	Refined grains
SD	Standard deviation
USDA	United States Department of Agriculture
VANISH	Volatile or Associated Negatively with Industrialised Societies of Humans
WG	Whole grains

## Appendix A

Table A1 displays food groups included in the industrialised and non-industrialised dietary components and the procedure used to derive the dietary gradient from the percentage of total energy contributed by each food group.

**Table A1:** Construction of the diet-ratio used in the multivariable analyses.

Dietary Component	Food Groups Included	Calculation
Industrialised component	Refined cereals, bakery products, sugar-sweetened beverages, fats, miscellaneous foods, alcoholic beverages	The percentage of total energy contributed by the selected food groups was summed to obtain the industrialised component. The resulting value was standardised as a z-score.
Non-industrialised component	Whole grains/cereals, fruits, vegetables, legumes, fish and seafood, eggs, nuts and seeds, dairy products, animal-source foods	The percentage of total energy contributed by the selected food groups was summed to obtain the non-industrialised component. The resulting value was standardised as a z-score.
Dietary gradient (diet-ratio)	—	diet-ratio = z-score (industrialised component) − z-score (non-industrialised component)

Note: “—, not applicable”.

Positive diet-ratio values indicate a greater relative contribution of industrialised foods to total dietary energy, whereas negative values indicate a greater relative contribution of non-industrialised foods.

The percentage contribution of each food group to total energy intake was calculated using a custom Stata script after nutrient estimation in Evalfinut 2.0. These values were used for constructing the diet-ratio.

**Table A2:** Sensitivity analyses evaluating abdominal pain as an additional covariate in beta- and alpha-diversity models.

Outcome	Primary Model	Model Adjusted for Abdominal Pain
PERMANOVA (reduced model)	R^2^ = 0.090; F = 1.22; *p* = 0.041	R^2^ = 0.109; F = 1.10; *p* = 0.182
db-RDA (reduced model)	F = 1.22; *p* = 0.041	F = 1.10; *p* = 0.182
Abdominal pain, db-RDA marginal term	__	*p* = 0.921
Shannon diversity: *Blastocystis* association	*p* = 0.018	*p* = 0.033
Shannon diversity: abdominal pain term	—	*p* = 0.207
Gini–Simpson diversity: *Blastocystis* association	*p* = 0.023	*p* = 0.040
Gini–Simpson diversity: abdominal pain term	—	*p* = 0.297
Observed richness: *Blastocystis* association	*p* = 0.024	*p* = 0.048
Observed richness: abdominal pain term	—	*p* = 0.083

Note: The primary beta-diversity model included diet-ratio, age, and BMI. Sensitivity analyses additionally included abdominal pain as a covariate. For alpha diversity, the adjusted models included *Blastocystis* colonisation, diet-ratio, age, BMI, sex, and abdominal pain. Alpha-diversity *p*-values for *Blastocystis* represent the association with colonisation before and after additional adjustment for abdominal pain, whereas *p*-values reported for abdominal pain represent the corresponding covariate term in the adjusted models. Shannon and Gini–Simpson diversity were the primary alpha-diversity indices; observed richness was included as a complementary sensitivity analysis. PERMANOVA and db-RDA results were based on the same Aitchison distance framework. Statistical significance was defined as *p* < 0.05. “—, not applicable”.

**Table A3:** Diagnostic evaluation of multivariable models used in the beta-diversity analyses.

**A. Homogeneity of Multivariate Dispersion (PERMDISP)**			
**Comparison**	**df**	**F**	***p*-Value**
Dietary tertiles	2	0.468	0.608
*Blastocystis* colonisation	1	1.999	0.157
Sex	1	0.541	0.499
**B. Multicollinearity Diagnostics (Variance Inflation Factors, VIF)**			
**Explanatory Variable**			**VIF**
Age			1.074
Sex			1.146
Body mass index (BMI)			1.187
*Blastocystis* colonisation			1.062
Diet-ratio			1.017

PERMDISP, Permutational Analysis of Multivariate Dispersions; VIF, Variance Inflation Factor; BMI, body mass index.

Homogeneity of multivariate dispersion was evaluated using PERMDISP prior to PERMANOVA. None of the grouping variables showed significant differences in dispersion (*p* > 0.05). Multicollinearity among explanatory variables was assessed using variance inflation factors (VIF). All VIF values were below 2, indicating negligible multicollinearity among predictors included in the multivariable model.

**Table A4:** Assessment of potential confounding variables considered in the multivariable analyses.

Potential Confounder	Assessment	Included in Final Multivariable Models	Rationale
Smoking status	Evaluated	No	Smoking prevalence was low in the study population (14.6%). Given its limited frequency and its lack of influence on the primary multivariable models, smoking was not retained as a covariate.
Abdominal pain	Evaluated	Sensitivity analyses only	Included as an additional covariate in sensitivity analyses because of its biological plausibility as a gastrointestinal factor potentially associated with gut microbial composition.
Alcohol intake	Considered	No	Alcohol intake constituted one component of the dietary exposure used to construct the diet-ratio. Separate adjustment was not performed to avoid potential overadjustment of the primary exposure.
Recent antibiotic use	Considered	No	Participants reporting antibiotic use within the three months preceding sample collection were excluded according to the study eligibility criteria. Therefore, recent antibiotic exposure was minimised by study design.
Probiotic consumption	Not available	No	Information regarding probiotic consumption was not collected; therefore, its potential influence on the gut microbiota could not be evaluated.
Physical activity (PAL category)	Evaluated	No	Physical activity was classified into three PAL categories (light, moderate and vigorous) exclusively to estimate total energy expenditure within the Goldberg–Black procedure for identifying implausible energy reporters. It was not collected as a quantitative measure of habitual physical activity and was therefore not included as an independent covariate in the multivariable ecological models.

Potential confounding variables considered during the statistical analysis, together with the rationale for their inclusion or exclusion from the final multivariable models.

Abdominal pain was evaluated through sensitivity analyses. Recent antibiotic exposure was controlled through the study eligibility criteria. Alcohol intake was considered part of the dietary exposure (diet-ratio), whereas information regarding probiotic consumption was not available.

**Table A5:** Distribution of *Blastocystis* subtypes among colonised participants.

Subtype Profile	*n*	% of Colonised Participants (*n* = 7)
ST3	1	14.3
ST3 + ST5	2	28.6
Not assigned to the targeted STS subtypes	4	57.1
Total	7	100

Subtype identification was performed by endpoint PCR using subtype-specific sequence-tagged site (STS) primers targeting ST1, ST2, ST3, ST4, ST5, and ST7. Four samples could not be assigned to any of the targeted subtypes despite previous confirmation of amplifiable DNA by β-globin PCR. Subtype analysis was performed on all *Blastocystis*-positive samples available for molecular characterisation.

**Table A6:** A. Model-level PERMANOVA statistics; B. Marginal term-wise PERMANOVA statistics.

**A. Model-Level PERMANOVA Statistics.**						
**Model**	**Raw R^2^**	**Adjusted R^2^**	**F**	***p*-Value**		
Full model	0.1387	0.0156	1.1268	0.0970		
Primary model	0.0901	0.0163	1.2206	0.0406		
*Blastocystis*-adjusted model	0.1139	0.0154	1.1567	0.0741		
Interaction model	0.1356	0.0121	1.0981	0.1523		
Primary model adjusted for abdominal pain	0.1085	0.0095	1.0955	0.1816		
**B. Marginal Term-Wise PERMANOVA Statistics**						
**Model**	**Term**	**df**	**SS**	**R^2^**	**F**	***p*-Value**
Full model	age	1	290.90	0.031	1.27	0.095
Full model	sex	1	229.10	0.024	1.00	0.454
Full model	bmi	1	298.96	0.032	1.31	0.067
Full model	*Blastocystis*	1	216.76	0.023	0.95	0.566
Full model	diet_ratio	1	261.60	0.028	1.14	0.213
Primary model	diet_ratio	1	272.25	0.029	1.19	0.156
Primary model	age	1	305.65	0.033	1.34	0.055
Primary model	bmi	1	253.00	0.027	1.11	0.268
*Blastocystis*-adjusted model	*Blastocystis*	1	220.41	0.023	0.96	0.526
*Blastocystis*-adjusted model	diet_ratio	1	269.05	0.029	1.18	0.178
*Blastocystis*-adjusted model	age	1	305.07	0.033	1.33	0.055
*Blastocystis*-adjusted model	bmi	1	269.16	0.029	1.18	0.171
Interaction model	age	1	283.22	0.030	1.23	0.119
Interaction model	bmi	1	298.37	0.032	1.30	0.070
Interaction model	diet_ratio: *Blastocystis*	1	200.62	0.021	0.87	0.714
Primary model adjusted for abdominal pain	diet_ratio	1	272.03	0.029	1.18	0.167
Primary model adjusted for abdominal pain	age	1	262.77	0.028	1.14	0.213
Primary model adjusted for abdominal pain	bmi	1	253.00	0.027	1.10	0.273
Primary model adjusted for abdominal pain	abdominal_pain	1	170.70	0.018	0.75	0.921

SS, sums of squares; R^2^, proportion of explained variance. Marginal term-wise tests were performed using adonis2 with by = “margin” and 9999 permutations. Each term was evaluated after adjustment for the remaining variables in the corresponding model.

**Table A7:** Alpha-diversity analyses according to *Blastocystis* colonisation.

**A. Descriptive Statistics and Unadjusted Effect Sizes**					
**Outcome**	**Non-Colonised, *n*; Median (IQR)**	**Colonised, *n*; Median (IQR)**	**Rank-Biserial r**	**95% CI**	***p*-Value**
Shannon	34; 2.510 (2.232–2.780)	7; 2.844 (2.748–2.885)	0.521	0.193 to 0.807	0.033
GiniSimpson	34; 0.837 (0.769–0.891)	7; 0.891 (0.880–0.897)	0.496	0.176 to 0.765	0.043
**B. Adjusted Multivariable Linear Regression Models**					
**Outcome**	**Variable**	**β (95% CI)**	***p*-Value**		
Shannon	*Blastocystis* colonisation (Yes vs. No)	0.398 (0.074 to 0.722)	0.018		
Shannon	Diet-ratio	0.104 (0.018 to 0.189)	0.019		
Shannon	Age	0.043 (−0.050 to 0.135)	0.353		
Shannon	Body mass index	0.027 (−0.001 to 0.054)	0.058		
Shannon	Sex (Male vs. Female)	−0.133 (−0.419 to 0.153)	0.352		
Gini-Simpson	*Blastocystis* colonisation (Yes vs. No)	0.075 (0.011 to 0.139)	0.023		
Gini-Simpson	Diet-ratio	0.022 (0.005 to 0.039)	0.012		
Gini-Simpson	Age	0.005 (−0.013 to 0.024)	0.553		
Gini-Simpson	Body mass index	0.005 (0.000 to 0.011)	0.058		
Gini-Simpson	Sex (Male vs. Female)	−0.047 (−0.103 to 0.010)	0.104		
**C. Overall Model Fit**					
**Outcome**	***n***	**R^2^**	**Adjusted R^2^**	**F**	***p*-Value**
Shannon	41	0.322	0.225	3.33	0.015
Gini-Simpson	41	0.353	0.260	3.81	0.007
**D. Leave-One-Out Sensitivity Analysis**					
**Outcome**	**Wilcoxon *p* Range**	**Adjusted β Range**	**Adjusted *p* Range**	**Direction Retained**	***n* Removed**
Shannon	0.022–0.078	0.331–0.493	0.006–0.057	Yes	7
Gini-Simpson	0.036–0.099	0.065–0.095	0.007–0.062	Yes	7

Notes. Alpha diversity was calculated at genus level from the original abundance matrix; no prevalence filter or centred log-ratio transformation was applied. Positive rank-biserial correlations indicate higher diversity among colonised participants. The 95% confidence intervals for these effect sizes were estimated from 10,000 bootstrap resamples performed separately within the colonised and non-colonised groups. The linear regression models included *Blastocystis* colonisation, diet-ratio, age, body mass index and sex. In the leave-one-out analysis, each colonised participant was removed in turn, and the unadjusted and adjusted associations were re-estimated.

**Table A8:** Sensitivity analyses supporting the construction and interpretation of the dietary gradient (diet-ratio).

**A. Correlations Among Dietary Components**							
**Comparison**				**Pearson r**		***p*-Value**	
Industrialised vs. non-industrialised components				−0.007		0.965	
Diet-ratio vs. industrialised component				0.710		<0.001	
Diet-ratio vs. non-industrialised component				−0.710		<0.001	
**B. Diet-Ratio Ranges and Group Sizes by Tertile**							
**Dietary Group**	**Tertile Definition (Diet-Ratio)**			**Diet-Ratio Range**		***n***	
Low industrialised	≤−0.520			−2.879 to −0.542		14	
Intermediate	>−0.520 to ≤0.159			−0.476 to 0.128		13	
High industrialised	>0.159			0.175 to 3.688		14	
**C. Energy Intake and Diet-Ratio According to Reporting Plausibility**							
**Analysis Sample**	***n***			**Energy, Median (IQR), kcal/day**		**Diet-Ratio, Median (IQR)**	
All participants	41			2435.0 (2134.0–2901.0)		−0.137 (−0.710 to 0.659)	
Plausible reporters	35			2334.0 (2113.5–2764.0)		−0.126 (−0.562 to 0.745)	
Implausible reporters	6			3780.0 (2890.0–4565.8)		−0.645 (−1.044 to −0.191)	
**D. Sensitivity of the Primary Multivariable Model**							
**Analysis Sample**	***n***	**PERMANOVA R^2^**	**Adjusted R^2^**	**F**	***p*-Value**	**db-RDA F**	***p*-Value**
All participants	41	0.090	0.016	1.22	0.041	1.22	0.041
Plausible reporters only	35	0.106	0.019	1.22	0.038	1.20	0.038

Notes. The lower and upper tertile boundaries were −0.520 and 0.159. Implausible energy reporters were identified using the Goldberg-Black method. Energy intake differed between plausible and implausible reporters (Wilcoxon *p* = 0.023), whereas the diet-ratio did not (*p* = 0.252). PERMANOVA and db-RDA were based on Euclidean distances calculated from the centred log-ratio-transformed genus abundance matrix and used 9999 permutations, with a fixed random seed (set.seed(123)) specified immediately before each permutation test.

**Table A9:** Spearman correlations between the dietary gradient (diet-ratio) and bacterial genera.

**Bacterial Genus**	**Spearman’s ρ**	***p*-Value**	**q-Value (FDR)**	**Direction**
*Flavonifractor*	−0.415	0.007	0.272	Low industrialised
*Ruminococcus gnavus* group	−0.389	0.012	0.272	Low industrialised
*Bacteroides*	−0.388	0.012	0.272	Low industrialised
*Ruthenibacterium*	−0.366	0.019	0.272	Low industrialised
*Paraprevotella*	−0.353	0.024	0.272	Low industrialised
UCG-005	0.345	0.027	0.272	High industrialised
*Streptococcus*	−0.340	0.030	0.272	Low industrialised
Lachnospiraceae UCG-010	−0.338	0.031	0.272	Low industrialised
*Caproiciproducens*	−0.331	0.035	0.272	Low industrialised
*Akkermansia*	−0.323	0.039	0.272	Low industrialised

Notes. Spearman correlations were calculated using centred log-ratio (CLR)-transformed genus-level abundances after applying the prevalence filter (>10% of samples). *p*-values were adjusted for multiple comparisons using the Benjamini-Hochberg false discovery rate (FDR) procedure. Positive correlations indicate association with the more industrialised end of the dietary gradient, whereas negative correlations indicate association with the less industrialised end. None of the associations remained statistically significant after FDR correction.

**Table A10:** Sequencing and DADA2 processing statistics for the 46 FACSA faecal samples included in the microbiome sequencing workflow.

**A. Overall Sequencing Summary**								
**Metric**	**Total Reads**		**Median Per Sample**		**Per-Sample Range**		**Overall Retention (%)**	
Input	6,864,121		152,395.5		88,884–184,330		—	
Filtered	5,048,440		111,806.0		17,203–139,874		73.55	
Denoised	4,869,998		106,628.5		16,309–136,793		70.95	
Merged	3,824,738		83,796.5		10,126–118,091		55.72	
Non-chimeric	438,699		9919.5		1890–11,437		6.39	
**B. Per-Sample Denoising Statistics**								
**Sample ID**	**Input**	**Filtered**	**Filtered (%)**	**Denoised**	**Merged**	**Merged (%)**	**Non-Chimeric**	**Non-Chimeric (%)**
220817-ESCTRL01	138,973	104,897	75.48	101,407	78,469	56.46	9555	6.88
220817-ESCTRL02	119,762	77,780	64.95	73,215	53,152	44.38	9271	7.74
220817-ESCTRL03	134,045	101,846	75.98	98,235	81,412	60.73	7125	5.32
220817-ESCTRL04	170,687	131,125	76.82	129,414	118,091	69.19	10,955	6.42
220817-ESCTRL05	122,542	90,966	74.23	86,953	62,896	51.33	8205	6.70
220817-ESCTRL06	154,419	118,004	76.42	114,549	95,501	61.85	10,875	7.04
220817-ESCTRL07	152,034	111,067	73.05	104,354	72,117	47.43	11,085	7.29
220817-ESCTRL08	184,330	136,719	74.17	131,026	97,585	52.94	11,437	6.20
220817-ESCTRL09	167,910	128,422	76.48	125,171	104,643	62.32	9612	5.72
220817-ESCTRL10	173,656	127,948	73.68	120,774	87,284	50.26	11,128	6.41
220817-ESCTRL11	152,757	115,062	75.32	111,230	92,525	60.57	7777	5.09
220817-ESCTRL12	173,100	127,546	73.68	122,541	88,932	51.38	11,306	6.53
220817-ESCTRL13	158,787	109,748	69.12	104,885	75,506	47.55	11,059	6.96
220817-ESCTRL14	166,755	126,644	75.95	123,790	105,382	63.20	9590	5.75
220817-ESCTRL15	88,884	17,203	19.35	16,309	10,126	11.39	1890	2.13
220817-ESCTRL16	165,870	125,475	75.65	120,435	87,686	52.86	9908	5.97
220817-ESCTRL17	150,935	109,652	72.65	105,037	82,073	54.38	10,506	6.96
220817-ESCTRL18	175,391	130,116	74.19	125,570	99,484	56.72	10,868	6.20
220817-ESCTRL19	151,393	111,920	73.93	106,533	78,229	51.67	9862	6.51
220817-ESCTRL20	176,136	139,874	79.41	136,793	117,507	66.71	10,529	5.98
220817-ESCTRL21	163,001	123,457	75.74	119,375	92,722	56.88	11,204	6.87
220817-ESCTRL22	157,025	118,769	75.64	114,743	92,641	59.00	10,418	6.63
220817-ESCTRL23	152,813	111,471	72.95	106,675	80,055	52.39	10,596	6.93
220817-ESCTRL24	156,494	116,287	74.31	112,144	91,839	58.69	9446	6.04
220817-ESCTRL25	147,003	105,653	71.87	101,633	80,281	54.61	8547	5.81
220817-ESCTRL26	156,394	115,782	74.03	112,654	91,397	58.44	10,117	6.47
220817-ESCTRL27	152,960	111,692	73.02	106,582	77,279	50.52	9793	6.40
220817-ESCTRL28	164,208	118,376	72.09	114,788	90,860	55.33	10,339	6.30
220817-ESCTRL29	161,990	118,851	73.37	115,013	89,321	55.14	10,583	6.53
220817-ESCTRL30	161,572	122,179	75.62	120,303	108,826	67.35	8974	5.55
220817-ESCTRL31	149,730	112,798	75.33	108,311	84,555	56.47	10,858	7.25
220817-ESCTRL32	157,363	119,244	75.78	115,299	91,378	58.07	11,402	7.25
220817-ESCTRL33	150,473	111,141	73.86	106,385	78,939	52.46	10,952	7.28
220817-ESCTRL34	151,902	112,975	74.37	108,748	83,350	54.87	9931	6.54
220817-ESCTRL35	147,965	105,765	71.48	99,703	66,665	45.05	10,959	7.41
220817-ESCTRL36	132,812	102,946	77.51	100,516	84,243	63.43	7040	5.30
220817-ESCTRL37	144,498	107,583	74.45	105,277	87,549	60.59	9185	6.36
220817-ESCTRL38	160,076	119,935	74.92	115,523	88,958	55.57	10,782	6.74
220817-ESCTRL39	117,466	89,403	76.11	86,792	69,928	59.53	7471	6.36
220817-ESCTRL40	134,440	100,084	74.45	97,204	76,295	56.75	8151	6.06
220817-ESCTRL41	137,752	101,432	73.63	98,934	79,552	57.75	7632	5.54
220817-ESCTRL42	130,817	94,416	72.17	88,963	62,370	47.68	9235	7.06
220817-ESCTRL43	127,559	97,130	76.15	94,589	80,557	63.15	10,302	8.08
220817-ESCTRL44	104,562	73,541	70.33	71,837	56,686	54.21	6369	6.09
220817-ESCTRL45	127,683	94,047	73.66	91,163	72,430	56.73	7957	6.23
220817-ESCTRL46	137,197	101,469	73.96	98,623	77,462	56.46	7913	5.77

Notes. Statistics were extracted from the QIIME 2 DADA2 denoising output for samples ESCTRL01–ESCTRL46. Percentages are relative to the input reads for each sample. Overall retention percentages were calculated from the summed counts across all 46 samples. DADA2 initially inferred 28,390 ASVs represented in the FACSA samples. ASV, amplicon sequence variant. Sequencing and DADA2 processing statistics are reported for all 46 successfully sequenced FACSA samples. Downstream integrated analyses were restricted to the 41 participants with complete microbiota, dietary and anthropometric data.

**Table A11:** Sensitivity analyses evaluating the influence of feature-table processing and sequencing depth on the main diversity findings.

Analysis	*n*	Shannon *p*-Value	Gini-Simpson *p*-Value	PERMANOVA R^2^	PERMANOVA F	PERMANOVA *p*-Value
Primary genus-level analysis	41	0.0331	0.0427	0.0901	1.2206	0.0406
Genus-level analysis excluding ESCTRL15	40	0.0390	0.0503	0.0926	1.2243	0.0358
De novo DADA2 ASV table (pre-closed reference)	41	0.0503	0.1822	0.0827	1.1117	0.1264
Rarefied to 4250 reads/sample, genus-level matched to primary analysis	40	0.0299	0.0462	0.0912	1.2045	0.0537

Notes. The primary analysis used the genus-level count table derived from the closed-reference workflow. ESCTRL15 was the lowest-depth sample (1890 post-DADA2 reads). The de novo sensitivity analysis used the DADA2 ASV table before closed-reference clustering. For the rarefaction sensitivity analysis, the feature table was rarefied without replacement to 4250 reads per sample and subsequently collapsed to the same genus-level representation used in the primary analysis; ESCTRL15 was therefore excluded because its sequencing depth was below the rarefaction threshold. Alpha-diversity *p*-values correspond to unadjusted comparisons according to *Blastocystis* colonisation status. PERMANOVA models included diet-ratio, age, and BMI and were evaluated using 9999 permutations. These analyses were conducted as sensitivity analyses and did not replace the prespecified primary genus-level analysis.
